# Guaianolide Sesquiterpene Lactones from Globe Artichoke (*Cynara scolymus* L.) Induce Nrf2-Associated Antioxidant Signaling

**DOI:** 10.3390/biomedicines14071589

**Published:** 2026-07-16

**Authors:** Preeti Kushwaha, Sualiha Afzal, Ritesh Raju, Xian Zhou, Gerald Münch

**Affiliations:** 1 Pharmacology Unit, School of Medicine, Western Sydney University, Campbelltown, NSW 2560, Australia; 2 NICM Health Research Institute, Western Sydney University, Westmead, NSW 2145, Australia

**Keywords:** *Cynara scolymus*, sesquiterpene lactones, Nrf2/ARE pathway, oxidative stress, glutathione

## Abstract

**Background/Objectives:** Oxidative and electrophilic stress-response pathways contribute to cellular resilience across various chronic diseases, including neurodegenerative, hepatic, and metabolic disorders. Pharmacological activation of the nuclear factor erythroid 2-related factor 2 (Nrf2)/antioxidant response element (ARE) pathway is a validated strategy to counteract oxidative damage, as evidenced by the clinical approval of dimethyl fumarate (DMF) and monomethyl fumarate (MMF) for relapsing forms of multiple sclerosis; both electrophilic compounds activate Nrf2 via covalent Keap1 (Kelch-like ECH-associated protein 1) modification. *Cynara scolymus* L. contains structurally related electrophilic metabolites; however, their contribution to Nrf2-associated signaling remains undefined. This study aimed to identify and characterize the constituents responsible for Nrf2-inducing activity and benchmark their potency against DMF and MMF. **Methods:** Bioactivity-guided fractionation combining Soxhlet extraction, Nrf2/ARE luciferase reporter screening, semi-preparative HPLC, and spectroscopic identification was employed. Functional validation included extracellular thiol quantification, H_2_O_2_ cytoprotection assays, and Western blot analysis of heme oxygenase-1 (HO-1). **Results:** The dichloromethane extract exhibited the highest Nrf2-inducing activity (54.4-fold). Fractionation yielded five guaianolide sesquiterpene lactones (**1**–**5**), four of which were active. The α-methylene-γ-lactone moiety was essential for activity. Aguerin B (**3**) exhibited the highest activity (39.14 ± 11.13-fold), while TBA analysis identified cynaropicrin (**2**) as the dominant extract-level contributor (62.9% of total activity). Notably, aguerin B (**3**) and cynaropicrin (**2**) induced greater reporter activity than DMF and MMF. Downstream pathway induction was confirmed by concentration- and time-dependent HO-1 upregulation, elevated extracellular glutathione and cysteinylglycine levels, and significant protection against H_2_O_2_-induced cytotoxicity without intrinsic toxicity. **Conclusions:** Guaianolide sesquiterpene lactones are the primary mediators of Nrf2-associated antioxidant signaling in *C. scolymus*. Cynaropicrin (**2**) exhibited stronger in vitro ARE-reporter induction than fumarates, supporting its relevance for further pharmacological investigation.

## 1. Introduction

Oxidative stress contributes to the pathology of a wide range of chronic and age-related diseases, including neurodegenerative conditions such as Alzheimer’s disease, Parkinson’s disease, and Friedreich’s ataxia [1], as well as hepatic and metabolic dysfunction [2]. Neurodegenerative diseases alone affect over 50 million people worldwide, according to WHO estimates. Although these conditions differ in clinical presentation and etiology, a unifying feature across these disorders is the persistent involvement of oxidative stress as a central driver of disease initiation and progression [3].

At the molecular level, oxidative stress arises from a disruption in redox homeostasis, characterized by an imbalance between the production of reactive oxygen species (ROS) and the capacity of endogenous antioxidant defense systems, contributing to the pathogenesis of numerous chronic diseases [4,5]. Excessive ROS generation leads to cumulative oxidative stress, which induces cellular damage, impairs DNA repair systems, and disrupts mitochondrial function, all of which are recognized as key contributors to accelerated aging and the development of chronic disorders [6].

The transcription factor nuclear factor erythroid 2-related factor 2 (Nrf2) is a master regulator of the cellular defense against oxidative and electrophilic stress. Under stress conditions or pharmacological activation, Nrf2 translocates to the nucleus and binds to the antioxidant response elements (AREs), thereby inducing the expression of a range of cytoprotective genes such as glutathione peroxidase (GPX), glutathione S-transferase (GST), glutamate-cysteine ligase (GCLC), NAD(P)H:quinone oxidoreductase 1 (NQO1), and heme oxygenase-1 (HO-1). These enzymes increase glutathione production, promote detoxification, and strengthen cellular resilience to oxidative injury [7,8,9].

Pharmacological activation of Nrf2 has therefore emerged as an anti-inflammatory and cytoprotective therapeutic strategy [10]. The recent clinical approval of omaveloxolone for Friedreich’s ataxia [11], dimethyl and monomethyl fumarate for multiple sclerosis [12,13] by the FDA, illustrates the translational potential of this pathway [14]. The Keap1-Nrf2 system is a broadly conserved cellular stress-response pathway, so Nrf2-inducing activity is typically first assessed in validated reporter and hepatic models, such as AREc32 and HepG2 [15], before progression to disease-relevant neuronal or glial systems.

Several plant-derived electrophilic metabolites, including sulforaphane from cruciferous vegetables such as *Brassica oleracea* (broccoli), curcumin from *Curcuma longa*, andrographolide from *Andrographis paniculata*, and carnosic acid from *Rosmarinus officinalis*, activate Nrf2 by covalently modifying reactive cysteine residues in its negative regulator Keap1 [16,17,18]. These findings emphasize the importance of natural products as valuable sources of redox-active scaffolds that can enhance endogenous antioxidant defenses.

Globe artichoke (*Cynara scolymus* L.) is a widely consumed vegetable and a traditional medicinal plant valued for its digestive, hepatoprotective, and antioxidant properties [19,20,21]. Although it contains several compounds, including phenolic acids, flavonoids, and sesquiterpene lactones, its ability to induce Nrf2-associated signaling and the specific molecular mechanisms underlying its cellular antioxidant effects remain insufficiently characterized.

The present study employs a bioassay-guided fractionation approach, coupled with an Nrf2/ARE-luciferase reporter system, to identify and characterize the Nrf2-inducing constituents of *C. scolymus*. Through chromatographic isolation and spectroscopic identification, we demonstrate that specific guaianolide-type sesquiterpene lactones potently induce Nrf2-associated signaling, increase glutathione levels, and attenuate H_2_O_2_-induced loss of cell viability, thereby providing a mechanistic basis for the traditional use of globe artichoke (GA) in promoting hepatic and metabolic health.

Importantly, this study moves beyond qualitative identification by quantitatively attributing Nrf2 activity to defined constituents using a combined potency-abundance framework.

## 2. Materials and Methods

### 2.1. Materials

Dried globe artichoke (*Cynara scolymus*) leaves material and derived extracts were provided by Integria Healthcare (Ballina, NSW, Australia). As the plant material was commercially sourced rather than field-collected, no herbarium voucher specimen was deposited. Batch identity and traceability were documented, and botanical identity and quality control were verified by the supplier in accordance with internal specifications and the British Pharmacopeia (BP) standards. A representative sample was retained for reference.

The AREc32 cells were provided by Professor Ronald C. Wolf, University of Dundee, UK, and the HepG2 cells were provided by Dr. Mitchell Low, NICM (National Institute of Complementary Medicine), Western Sydney University, Australia. AREc32 cells (a MCF7-derived ARE-luciferase reporter line) were used as a sensitive, established ARE-luciferase reporter model for initial detection of Nrf2/ARE pathway induction, while HepG2 cells served as a complementary hepatic model for validation of downstream protein responses.

Dulbecco’s Modified Eagle’s Medium (DMEM), streptomycin, trypan blue, and resazurin sodium salt were purchased from Sigma-Aldrich (Castle Hill, NSW, Australia). GIBCO Fetal bovine serum (FBS) and glutamine were purchased from Life Technologies (Mulgrave, VIC, Australia). Acetonitrile, n-hexane, dichloromethane, ethyl acetate, ethanol, and methanol, and all other chemicals were supplied by Merck Pty. Ltd. (Kilsyth, VIC, Australia). D-luciferin-potassium salt and Coenzyme A were purchased from Sapphire Bioscience (Redfern, NSW, Australia).

### 2.2. Methods

#### 2.2.1. Extraction of Globe Artichoke Leaf Powder

Approximately 240 g of globe artichoke (*Cynara scolymus*) leaves powder was filled into the thimbles of an accelerated solvent extraction system (Buchi B-811, Flawil, Switzerland) and extracted (for 4 × 30 min cycles) using six solvents at the indicated temperature [n-hexane (68.7 °C), dichloromethane (39.6 °C), ethyl acetate (77.1 °C), methanol (64.7 °C), ethanol (78.37 °C), and water (100 °C)]. The volume of the extracts was reduced to 2–4 mL using a rotary evaporator, and the extracts were then completely dried with nitrogen.

#### 2.2.2. Isolation of Fractions by Semipreparative HPLC

From the most active dichloromethane (DCM) 8.0 g extract, approximately 2.0 g was resuspended in MeOH and was later subjected to reverse-phase HPLC (Agilent 1260 Infinity II series) using a semi-preparative Eclipse XDB-C18 column (10 × 250 mm, 10 μm). The flow rate was set at 2 mL/min, and the solvent system used was from 10 to 100% MeOH/H_2_O (0.01% FA) over 55 min, and held at 100% MeOH for 10 min, and then equilibrated back to 10% MeOH from 65 to 66 min and maintained 10% MeOH for 4 min for the reconditioning of the column.

#### 2.2.3. Culture of AREc32 (Luciferase Reporter) Cells

AREc32 cells were cultured in 75 cm^2^ flasks (SARSTEDT, Mawson Lakes, SA, Australia) using DMEM supplemented with 10% fetal bovine serum (FBS), 100 μg/mL penicillin-streptomycin, and 2 mM L-glutamine. The cells were maintained in a humidified incubator at 37 °C with 5% CO_2_, and the culture medium was replenished every 3–4 days. Upon reaching full confluence, the cells were detached using trypsin. The resulting cell suspension was then concentrated by centrifugation at 1500 rpm for 5 min and resuspended in a small volume of fresh DMEM supplemented with 1% antibiotics and 10% FBS. Cell density was determined using a Neubauer counting chamber, and the concentration was adjusted with DMEM (containing 1% antibiotics and 10% FBS) to 1 × 10^6^ cells per 100 μL. A total of 100 μL of this cell suspension was subsequently dispensed into each well of a 96-well plate. The plates were incubated at 37 °C with 5% CO_2_ for 48 h prior to the commencement of activation experiments.

#### 2.2.4. Activation of AREc32 Cells

After 48 h, the medium was removed from each well, and 100 μL of the respective dilutions of extracts/pure compound in DMEM containing 0.1% FBS was added to each well to activate Nrf2-induced luciferase transcription. The maximum concentrations used were 100 μg/mL for direct extracts and 50 μM for tert-butylhydroquinone (tBHQ) (dissolved in 0.5% ethanol), with a minimum of 11 doses tested. Each compound was serially diluted starting from a concentration of 100 μg/mL. Non-activated cells (exposed to 0.1% FBS in DMEM alone) served as blanks, while cells activated with tBHQ (50 μM) were used as a positive control.

#### 2.2.5. Determination of Cell Viability by the Alamar Blue Assay

After 24 h of treatment, the cell culture supernatant was transferred to a new 96-well plate and stored at −20 °C for thiol measurement. To assess cell viability, 100 μL of Alamar Blue solution (10% resazurin in DMEM with 0.1% FBS) was added to each well of the original plate, and the plate was incubated at 37 °C for 1 h. Fluorescence intensity was measured using a microplate reader (excitation: 530 nm, emission: 590 nm) and expressed as a percentage relative to non-activated cells.

#### 2.2.6. Determination of Nrf2 Reporter Activity with the Luciferase Assay

Following the removal of Alamar Blue, AREc32 cells were washed with 1× phosphate-buffered saline (PBS), the PBS was aspirated, and 20 μL of lysis buffer (Tris-HCl: 1.705%, Base: 0.508%, 5M NaCl: 1.5%, 1M MgCl_2_: 0.3%, Triton X-100 pure liquid: 0.75%) was added to each well. After 15 min of shaking and 20 min of freezing at 20 °C, 15 μL of cell lysate was transferred into the white plates. Cell lysates (15 μL) received 100 μL of luciferase assay reagent at the following concentrations: 1 M DTT (3%), 100 mM ATP (0.45%), D-Luciferin (0.525%), and 10 mM CoA (6%) at room temperature. Nrf2 activity was measured in relative luminescence unit (RLU) and expressed as fold change relative to the non-activated control (concentration = 0, cells with medium only).

#### 2.2.7. Thiol Measurement by High-Performance Liquid Chromatography

A Dionex HPLC system, comprising a WPS-3000 automated sample injector, an Ultimate 3000 pump, an ACC-3000 autosampler and column compartment, and an FLD-3100 fluorescence detector, was used. The system was equipped with a reverse-phase RP-18 endcapped column (150 × 4.6 mm, 5 μm) protected by a SecurityGuardC18 cartridge (4.0 × 3.0 mm) in a Security Guard cartridge holder (Phenomenex, Torrance, CA, USA). Chromeleon 7.0 Chromatography Data System (Dionex, Sunnyvale, CA, USA) was used to control the instruments, acquire data, and quantify peak areas. Thiol detection was performed as described previously [22,23].

#### 2.2.8. H_2_O_2_-Induced Cell Injury Assay

To investigate the protective effects of the four isolated artichoke compounds, AREc32 cells were pre-treated with increasing concentrations of the Nrf2-active compounds (0.78–100 μg/mL) and an optimized concentration of tBHQ (50 μM) for 24 h, followed by exposure to H_2_O_2_ (2.5 mM) for 24 h. Cell viability was subsequently assessed using the Alamar Blue assay, as described previously in Section 2.2.5.

#### 2.2.9. Western Blot Analysis

For the Western blot experiment, HepG2 cells were selected because they are a well-characterized, Nrf2/Keap1-responsive hepatic cell line widely used to study the induction of cytoprotective and detoxification genes [15]. The cells were cultured in T75 flasks at 37 °C in a humidified atmosphere containing 5% CO_2_ until ~95% confluence. Cells were treated with control medium (0.1% FBS in DMEM) or cynaropicrin (**2**) (3.125, 6.25, and 12.5 μg/mL; DMSO ≤ 0.1%, *w*/*v*) for 4, 6, 12, and 24 h. Cells were harvested by centrifugation (500× *g*, 5 min, 4 °C), and pellets were collected for protein extraction.

Cell pellets were lysed on ice in RIPA buffer (25 mM Tris-HCl, 150 mM NaCl, 1% NP-40, 1% sodium deoxycholate, 0.1% SDS) supplemented with protease inhibitor cocktail (1%, Cell Signaling Technology, Danvers, MA, USA) for 30 min with intermittent vortexing. Lysates were clarified by centrifugation (14,000× *g*, 15 min, 4 °C), and supernatants were collected. Protein concentrations were determined using the Pierce BCA Protein Assay Kit (Thermo Fisher Scientific, Scoresby, VIC, Australia). Equal amounts of protein (20 μg) were separated on 10–15% gradient SDS-PAGE gels (Bio-Rad, South Granville, NSW, Australia) and transferred onto nitrocellulose membranes (100 V, 2 h).

Membranes were blocked with 5% (*w*/*v*) skim milk in TBST (TBS containing 0.1% Tween-20) for 1 h at room temperature and incubated overnight at 4 °C with primary antibodies against HO-1 (1:1000, Cat. No. 26416S) and glyceraldehyde 3-phosphate dehydrogenase (GAPDH) (1:2000, Cat. No. 2118). After washing, membranes were incubated with HRP-conjugated anti-rabbit secondary antibody (1:10,000, Cat. No. 7074, Cell Signaling Technology, Danvers, MA, USA) for 2 h at room temperature.

Protein bands were detected using SuperSignal West Pico PLUS ECL reagent (Thermo Fisher Scientific, Scoresby, VIC, Australia) and imaged using an iBright CL750 system (Thermo Fisher Scientific, Scoresby, VIC, Australia). Band intensities were quantified using ImageJ version 1.54p (NIH, Bethesda, MD, USA), normalized to GAPDH, and expressed relative to the control. All Western blot experiments were performed in at least three independent biological replicates. Exposure times were adjusted to ensure signal detection within the linear range.

#### 2.2.10. NMR Spectra

NMR spectra were recorded on a Bruker Ascend 600 MHz spectrometer (Bruker Biospin GmbH, Bremen, Germany), in the solvents indicated and referenced to residual ^1^H signals in deuterated solvents.

#### 2.2.11. Mass Spectrometry

HRMS (High Resolution Mass Spectrometry) was carried out using a Waters SYNAPT G2-Si mass spectrometer operating in the positive ESI mode as described previously [24].

#### 2.2.12. Statistical Analysis

Statistical analysis was performed using GraphPad Prism 11.0 (GraphPad Software, San Diego, CA, USA). Data are presented as mean ± standard deviation (SD), based on at least three independent biological experiments, each performed in technical triplicate. Group differences were assessed using one-way or two-way analysis of variance (ANOVA), followed by Dunnett’s multiple comparison test where appropriate. For all statistical tests, a *p*-value < 0.05 was considered statistically significant. * *p* < 0.05, ** *p* < 0.01, *** *p* < 0.001, **** *p* < 0.0001 vs. control (concentration = 0, cells with medium only).

## 3. Results

### 3.1. Determination of the Degree of Nrf2/ARE Reporter Induction by a Commercial Globe Artichoke Extract

**Figure 1:**
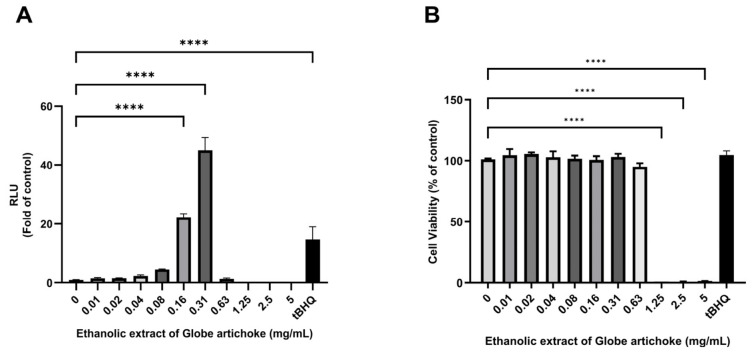
Nrf2/ARE reporter induction and cell viability following treatment with ethanolic extract of globe artichoke. AREc32 cells were exposed to the serial dilutions of the extract for 24 h. (**A**) Reporter activity and (**B**) cell viability are shown as ± SD of 3 individual experiments in triplicate. Significance was assessed by one-way ANOVA, **** *p* < 0.0001 vs. control (concentration = 0).

To identify edible plants with strong Nrf2/ARE pathway activation and minimal cytotoxicity, a panel of commercial edible and medicinal extracts was screened using AREc32 (Nrf2 luciferase) reporter cells. AREc32 cells provide a sensitive mechanistic reporter system, although they do not fully model tissue-specific pharmacodynamics. In addition to our recently published similar results on Nrf2 activators from Valerian root [24], the ethanolic extract of globe artichoke leaves emerged as one of the most potent active extracts in this screening panel, increasing luminescence by approximately 40-fold relative to non-activated control cells. Maximal induction occurred at an extract concentration of 0.31 mg/mL, with no detectable cytotoxicity up to 0.63 mg/mL (Figure 1A,B).

### 3.2. Sequential Extraction of the Globe Artichoke Powder

**Figure 2:**
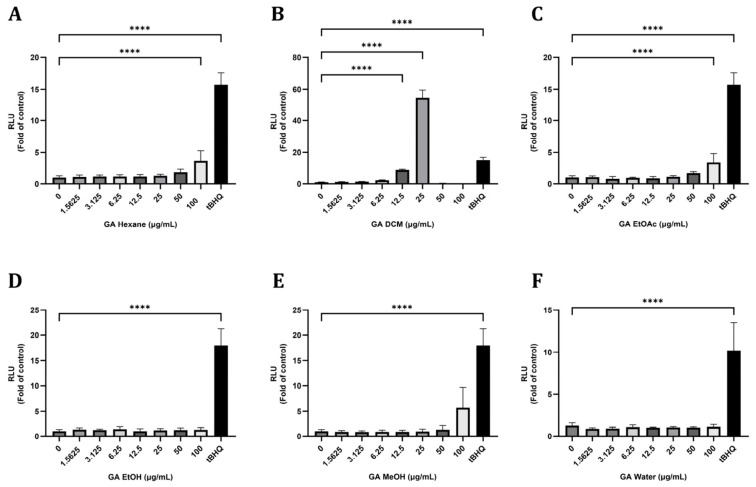
Nrf2/ARE reporter induction by sequential extracts of globe artichoke. AREc32 cells were treated with serial dilutions of each solvent extract (μg/mL) and tBHQ (50 μM) for 24 h. Data are presented as the mean ± SD of 3 individual experiments in triplicate. Significance was assessed by one-way ANOVA, **** *p* < 0.0001 vs. control (concentration = 0). Panels: (**A**) n-hexane, (**B**) DCM, (**C**) EtOAc, (**D**) EtOH, (**E**) MeOH, (**F**) water.

Sequential Soxhlet extraction using solvents of increasing polarity was conducted to distribute Nrf2-relevant bioactive compounds across distinct fractions. The DCM extract exhibited the highest potency, yielding a 54.4-fold increase in Nrf2 activity at 25 μg/mL without cytotoxicity (Table 1, Figure 2B). In contrast, the remaining fractions produced only modest induction (1–4-fold) (Figure 2A,C–F), indicating that Nrf2-inducing activity was concentrated in the DCM extract. Based on these screening results, the active DCM extract was selected for subsequent chromatographic purification to isolate Nrf2/ARE-inducing constituents. Corresponding cell viability data for all sequential extracts are presented in Figure S1.

### 3.3. HPLC Fractionation and Compound Identification

**Figure 3:**
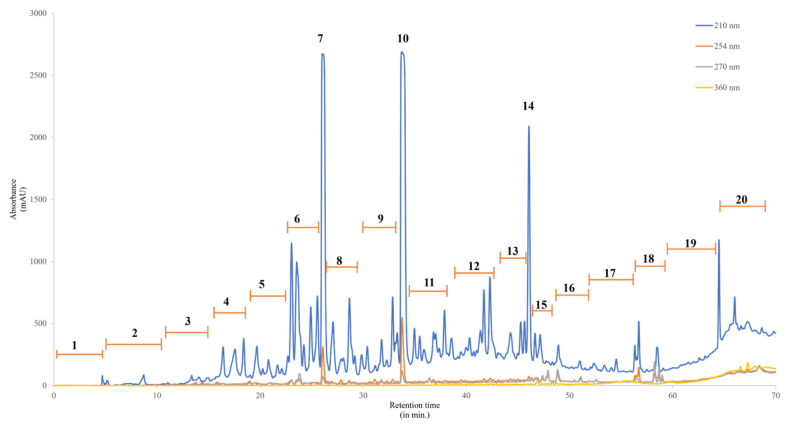
HPLC-DAD chromatogram of the DCM extract from globe artichoke (210 nm). Semi-preparative HPLC chromatogram of the DCM extract showing resolution into multiple subfractions. Chromatographic separation was achieved on an XDB-C18 column (10 × 250 mm, 10 μm) using a linear gradient from 10 to 100% MeOH/H_2_O containing 0.01% formic acid over 55 min at 2.0 mL/min.

**Figure 4:**
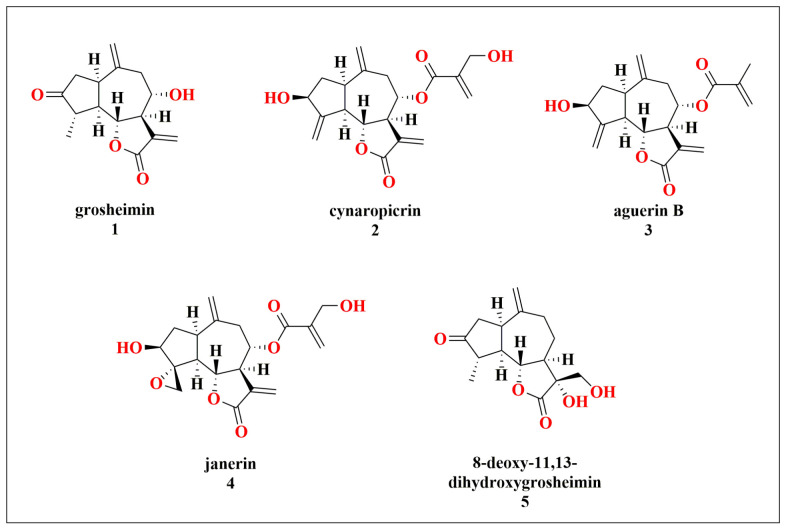
Chemical structures of sesquiterpene lactones (**1**–**5**) isolated from globe artichoke.

The active DCM extract was fractionated by semi-preparative HPLC, yielding 20 subfractions (Figure 3). Eight subfractions showed marked Nrf2-inducing activity, with reporter activation ranging from 31- to 64-fold (Table 2; Figure S2). Further purification led to the isolation of five guaianolide sesquiterpene lactones: grosheimin (1; Fr. 7, t_r_ = 25.5 min) [25], cynaropicrin (2; Fr. 10, t_r_ = 33.1 min) [26], aguerin B (3; Fr. 14, t_r_ = 45.5 min) [27], janerin (4; Fr. 6e, t_r_ = 7.7 min) [28], and 8-deoxy-11,13-dihydroxygrosheimin (5; Fr. 6g, t_r_ = 5.9 min) [29] (Figure 4). The analytical HPLC profile of GA_DCM fraction 6 used for secondary purification is shown in Figure S3.

**Figure 5:**
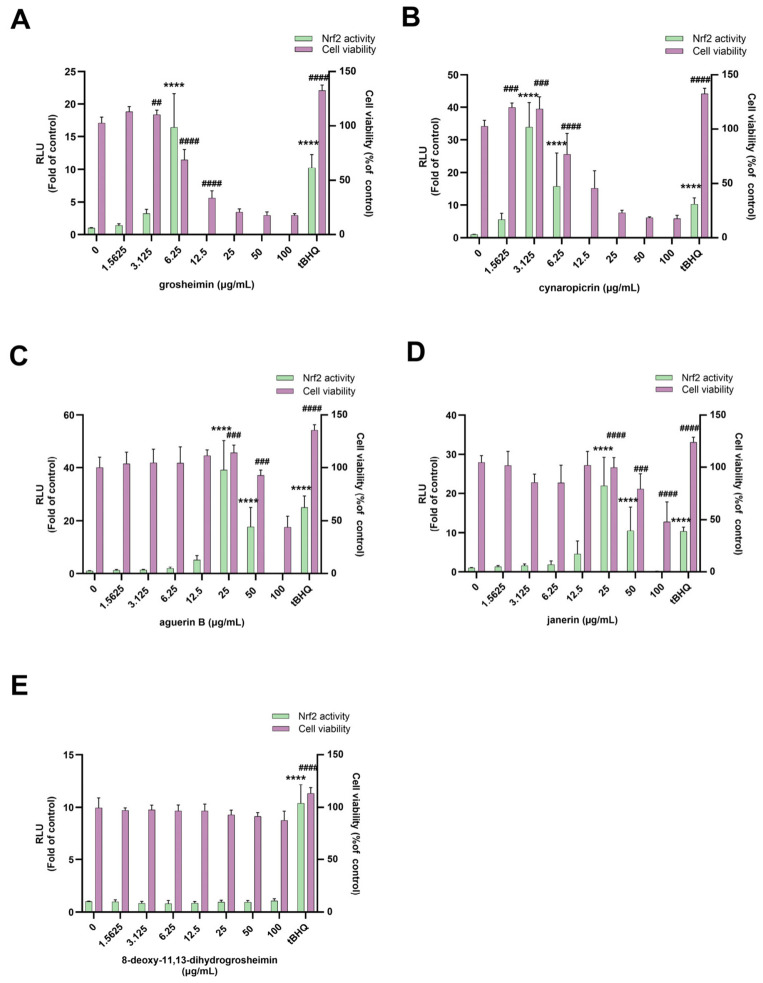
Nrf2/ARE reporter induction and cell viability of pure compounds isolated from the DCM extract of globe artichoke. AREc32 cells were exposed to serial dilutions of compounds and tBHQ (50 μM) for 24 h. Data are presented as the mean ± SD of 3 individual experiments in triplicate. Significance was compared by one-way ANOVA analysis; **** *p* < 0.0001 vs. control (concentration = 0) for Nrf2 activity; ^##^ *p* < 0.01, ^###^ *p* < 0.001, and ^####^ *p* < 0.0001 vs. control (concentration = 0) for cell viability. Panel: (**A**) grosheimin, (**B**) cynaropicrin, (**C**) aguerin B, (**D**) janerin, and (**E**) 8-deoxy-11,13-dihydroxygrosheimin.

Among the isolated compounds, aguerin B (**3**) showed the strongest Nrf2-inducing activity (39.14 ± 11.13-fold), followed by cynaropicrin (**2**) (33.94 ± 7.48-fold) and janerin (**4**) (21.96 ± 7.3-fold), whereas grosheimin (**1**) produced a more modest response (16.45 ± 5.18-fold) at the same concentration. In contrast, 8-deoxy-11,13-dihydroxygrosheimin (**5**) was inactive across the tested concentration range (Figure 5). These data indicate that an intact α-methylene-γ-lactone motif is associated with Nrf2/ARE reporter induction, while additional substitution at C-8 may further modulate activity. Except for compound **1**, no significant reduction in cell viability was observed at concentrations corresponding to maximal Nrf2 reporter induction (Figure 5). The purity of all compounds was confirmed by LC–MS analysis and was found to be >90% (Figure S4). Full spectroscopic data (^1^H NMR and HRMS) are reported and are consistent with previously reported literature values (Figures S5–S14).

### 3.4. Contribution of Principal Compounds to the Total Nrf2 Bioactivity

The relative contribution of individual constituents and fractions to the Nrf2-inducing activity of the dichloromethane extract was assessed by evaluating the isolated fractions and compounds in terms of both potency and relative abundance. EDV_50_ and total bioactivity (TBA) were then calculated to quantify each compound’s contribution to the overall activity, using an approach previously introduced by our group [30,31]. Here, EDV_50_, used as a measure of potency, represents the concentration required to achieve 50% of maximal Nrf2 reporter induction, while total bioactivity (TBA) was calculated by combining compound potency with relative abundance. This analysis revealed that cynaropicrin (**2**) accounted for 62.9% and grosheimin (**1**) for 11.3% of the total extract activity, together explaining more than two-thirds of the overall potency (Table 3).

### 3.5. Effects of Active Compounds on Glutathione and Related Metabolites

**Figure 6:**
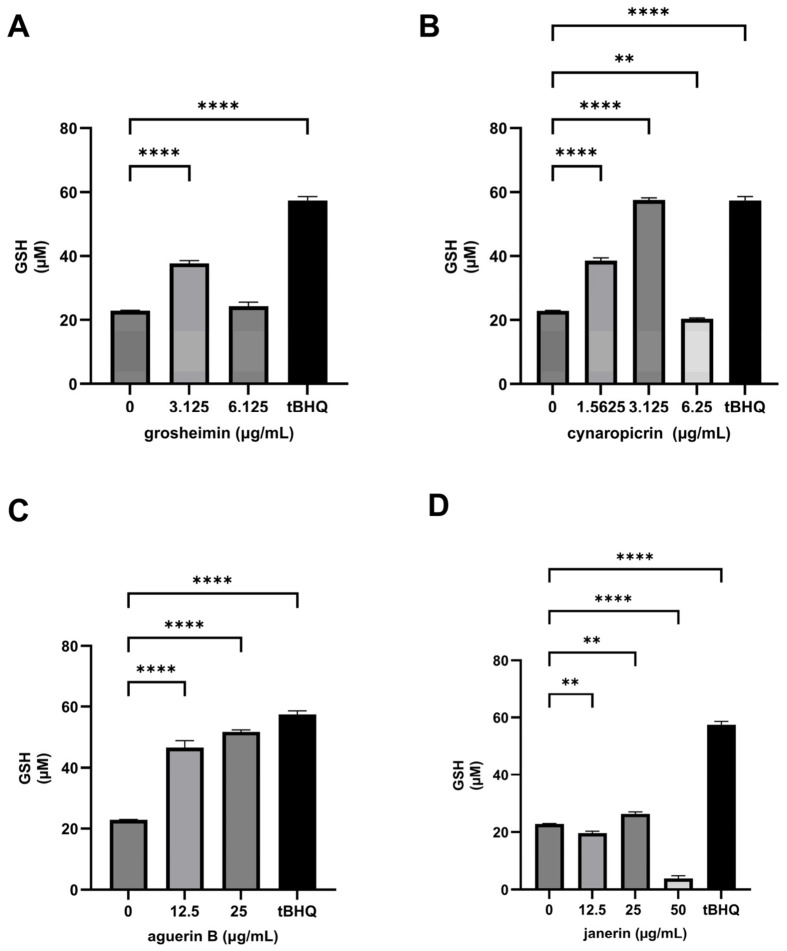
Sesquiterpene lactones induce extracellular GSH levels in AREc32 cells. Cells were treated with serial dilutions of isolated compounds and tBHQ (50 μM) for 24 h, and extracellular GSH was measured by HPLC with fluorescence detection. Data are presented as mean ± SD of 3 individual experiments. Significance was assessed by one-way ANOVA; ** *p* < 0.01, **** *p* < 0.0001 vs. control (concentration = 0). Panel: (**A**) grosheimin, (**B**) cynaropicrin, (**C**) aguerin B, and (**D**) janerin.

**Figure 7:**
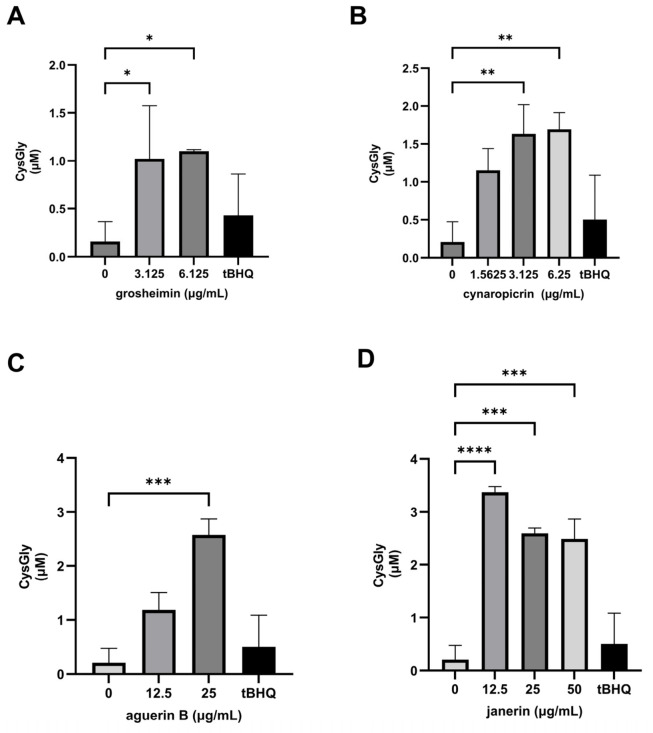
Sesquiterpene lactones induce extracellular CysGly in AREc32 cells. Cells were treated with serial dilutions of active compounds and tBHQ (50 μM) for 24 h, and extracellular CysGly was measured by HPLC with fluorescence detection. Data are presented as mean ± SD of 3 individual experiments. Significance was determined by one-way ANOVA analysis; * *p* < 0.05, ** *p* < 0.01, *** *p* < 0.001, **** *p* < 0.0001 vs. control. Panel: (**A**) grosheimin, (**B**) cynaropicrin, (**C**) aguerin B, and (**D**) janerin.

Cells treated with the Nrf2-inducing sesquiterpene lactones exhibited altered extracellular thiol metabolism, evidenced by increased concentrations of glutathione (GSH) and cysteinylglycine (CysGly) measured by HPLC with fluorescence detection (Figure 6 and Figure 7). Basal extracellular GSH levels (22.65 ± 0.14 μM) were elevated to 2.5-fold (57.46 ± 1.17 μM) following treatment with the positive control tBHQ. Treatment with cynaropicrin (**2**) elevated extracellular GSH levels to approximately 57.60 ± 0.66 μM at 3.125 μg/mL, matching the response observed with the positive control. Similar but less pronounced upward trends were noted for aguerin B (**3**) (51.76 ± 0.64 μM) at 25 μg/mL. Grosheimin (**1**) produced a more moderate elevation (37.69 ± 0.87 μM), while janerin (**4**) induced only a modest increase at its most effective concentration. Consistent with these findings, extracellular CysGly levels; indicative of accelerated glutathione catabolism and systemic thiol turnover were significantly increased relative to control values (~0.2 μM), reaching ~1.1 μM, ~1.7 μM, ~2.6 μM, and ~3.3 μM following treatment with grosheimin (**1**), cynaropicrin (**2**), aguerin B (**3**), and janerin (**4**), respectively. Collectively, these data demonstrate that the sesquiterpene lactones identified as Nrf2/ARE-inducing constituents promote extracellular thiol accumulation.

### 3.6. Cytoprotective Potential of the Bioactive Compounds Against Hydrogen Peroxide

**Figure 8:**
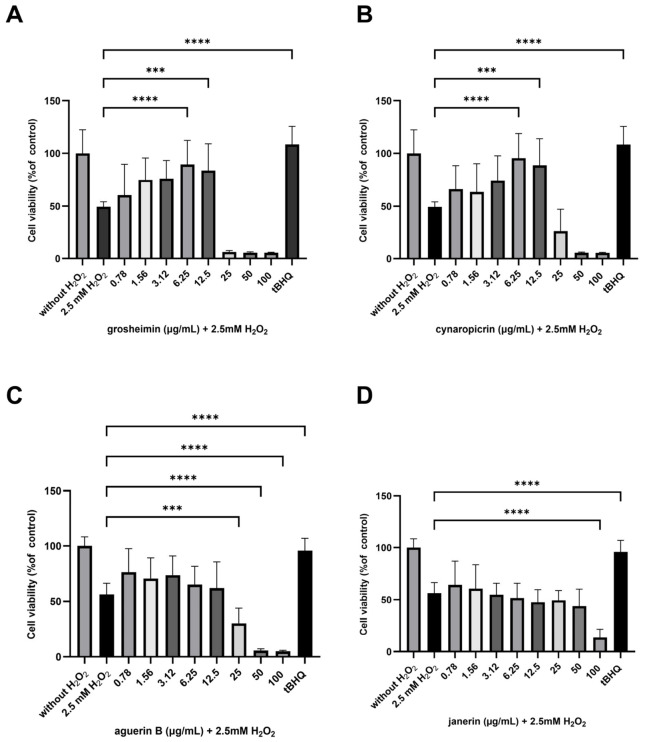
Sesquiterpene lactone Nrf2/ARE inducers attenuate hydrogen peroxide-induced loss of cell viability. AREc32 cells were treated with serial dilutions of active compounds for 24 h, followed by exposure to 2.5 mM H_2_O_2_ for 24 h. Cell viability was assessed by the Alamar Blue assay. Data are presented as mean ± SD of 3 individual experiments in triplicate. Significance was determined by one-way ANOVA analysis; *** *p* < 0.001, **** *p* < 0.0001 vs. 2.5 mM H_2_O_2_. Panel: (**A**) grosheimin, (**B**) cynaropicrin, (**C**) aguerin B, and (**D**) janerin.

Hydrogen peroxide is primarily detoxified by a complex network of enzymes, including glutathione peroxidase and catalase. Their activity relies on the cellular glutathione pool; thus, an efficient Nrf2-driven antioxidant system protects cells from H_2_O_2_-induced oxidative damage and cell death [32]. To assess functional cytoprotection, AREc32 cells were pretreated with isolated compounds for 24 h before exposure to 2.5 mM H_2_O_2_ (Figure 8). Cynaropicrin (**2**) and aguerin B (**3**) demonstrated the most potent effects, significantly attenuating H_2_O_2_-induced loss of cell viability across a broad concentration range of 0.78–12.5 μg/mL (Figure 8B,C), while grosheimin (**1**) and janerin (**4**) provided moderate protection (Figure 8A,D). These cytoprotective trends broadly align with the optimal thresholds of enhanced thiol metabolism, confirming that Nrf2-mediated glutathione biosynthesis and functional adaptation mediate the observed cellular defense against severe oxidative stress.

### 3.7. Effect of Cynaropicrin (***2***) on Nrf2-Mediated Expression of HO-1

Further characterization of downstream Nrf2 signaling focused on cynaropicrin (**2**), selected for its optimal combination of potent Nrf2 activity and adequate isolation yield for experimental evaluation. We investigated its ability to induce a well-established downstream target, heme oxygenase-1 (HO-1). HO-1 is a canonical Nrf2-dependent enzyme and a widely used biomarker of pathway activation, where its upregulation as a phase II detoxification enzyme reflects an antioxidant cellular response downstream of Nrf2 signaling [33].

**Figure 9:**
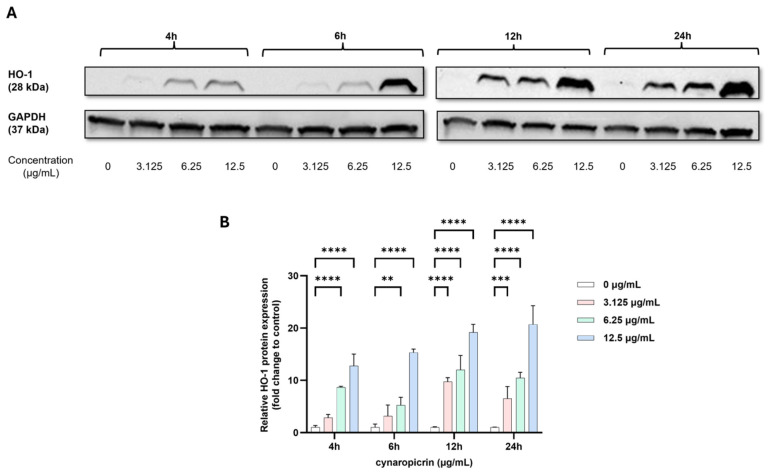
Cynaropicrin (**2**) induces HO-1 expression in HepG2 cells. (**A**) Relative protein expression of heme oxygenase 1 (HO-1) following the treatment of cynaropicrin (**2**). (**B**) Densitometric quantification of HO-1/GAPDH ratios using ImageJ. Data are presented as mean ± SD (*n* = 3 individual experiments). Statistical analysis was determined using two-way ANOVA with Dunnett’s multiple comparisons test; ** *p* < 0.01, *** *p* < 0.001, **** *p* < 0.0001 vs. control (concentration = 0).

Densitometric analysis of band intensities normalized to GAPDH confirmed these HO-1 expression changes. Cynaropicrin (**2**) induced HO-1 protein expression in a concentration- and time-dependent manner. After 4 h, significant increases were observed at 6.25 and 12.5 μg/mL compared to the control (concentration = 0; cells with media only), with the highest concentration producing a ~13-fold induction, whereas 3.125 μg/mL had no effect. HO-1 expression increased progressively over time, reaching ~15–18-fold above control at 12 h and ~18–20-fold at 24 h at 12.5 μg/mL, indicating sustained induction and protein stability. Two-way ANOVA showed significant effects of concentration and time, as well as their interaction. Dunnett’s post hoc test confirmed significant increases at 6.25 and 12.5 μg/mL across all time points, with no significant change at 3.125 μg/mL (Figure 9).

### 3.8. Comparative Evaluation of Cynaropicrin (***2***) and Aguerin B (***3***) Against FDA-Approved Nrf2 Activators DMF and MMF

**Figure 10:**
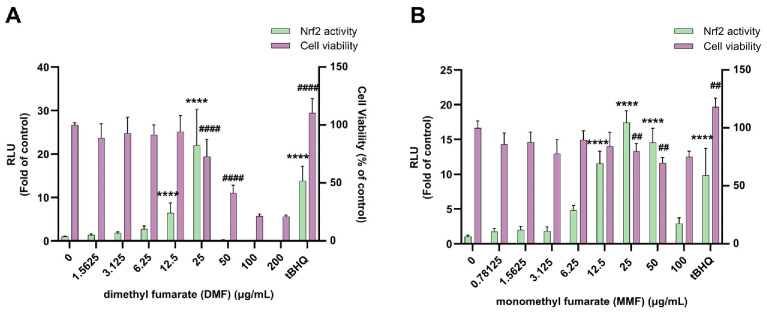
Nrf2/ARE reporter induction and cell viability of clinically approved fumarates. AREc32 cells were treated with serial dilutions of (**A**) DMF and (**B**) MMF, for 24 h. Data are presented as mean ± SD of 3 individual experiments in triplicate. **** *p* < 0.0001 vs. control (concentration = 0) for Nrf2 activity; ^##^ *p* < 0.01, and ^####^ *p* < 0.0001 vs. control (concentration = 0) for cell viability, compared by one-way ANOVA.

The Nrf2/ARE-inducing capacities of cynaropicrin (**2**) and aguerin B (**3**) were directly compared to the FDA-approved Nrf2 activators dimethyl fumarate (DMF) and monomethyl fumarate (MMF). The AREc32 cell-based luciferase reporter assay revealed that both compounds exhibited markedly superior Nrf2 reporter induction profiles compared to reference fumarates, acting at significantly lower effective concentrations (Figure 5). Specifically, the maximum reporter induction achieved by DMF (21.98 ± 8.27-fold) and MMF (17.42 ± 1.71-fold) required a high concentration of 25 μg/mL. Notably, cynaropicrin (**2**) exhibited higher fold-induction compared to the reference fumarates at a much lower concentration of 3.125 μg/mL. Similarly, at an equivalent dose of 25 μg/mL, aguerin B (**3**) more than doubled the reporter induction of MMF (Figure 10).

## 4. Discussion

The study demonstrates that Nrf2-inducing activity in globe artichoke (*Cynara scolymus*) is confined to a discrete, chemically defined class of metabolites rather than being a diffuse property of the plant matrix. Sequential solvent extraction across a range of increasing polarities revealed that the dichloromethane (DCM) fraction concentrates the Nrf2-inducing constituents. This observation is consistent with polarity-dependent solubility during Soxhlet extraction. Moderately lipophilic guaianolide sesquiterpene lactones dissolve efficiently in DCM, while more polar phenolic acids and flavonoids require methanol, ethanol, or water for extraction. This aligns with the established polarity-dependent partitioning of these compounds in artichoke extracts [34]. As extraction proceeded sequentially by increasing polarity, the sesquiterpene lactone-rich fractions were obtained first. This process may have already depleted much of the plant material’s electrophilic constituents prior to the methanolic and aqueous extraction steps. The non-polar n-hexane fraction exhibited minimal activity, indicating that Nrf2/ARE induction operates within a specific lipophilicity window rather than simple non-polarity.

Globe artichoke is rich in phenolic acids and flavonoids (e.g., chlorogenic acid, luteolin), which are predominantly found in the aqueous and methanolic fractions. The near-absence of ARE-luciferase activity in these fractions suggests that these constituents contribute minimally to direct ARE-reporter induction. This is consistent with prior evidence that the antioxidant capacity of dietary flavonoids, as measured by direct free-radical scavenging assays, does not reliably predict their ability to induce Nrf2/ARE-luciferase reporter activity, indicating that these represent mechanistically distinct properties [35]. Instead, the plant’s electrophilic signaling capacity appears to reside in a chemically distinct part of its metabolome. This distinction does not negate the biological relevance of artichoke polyphenols, which likely mediate antioxidant effects via complementary radical-scavenging mechanisms rather than direct engagement of the Nrf2/ARE pathway. This distinction is also important for extract standardization, as it helps ensure a consistent and mechanistically defined pharmacological effect.

To our knowledge, this is the first study to quantitatively attribute Nrf2-inducing activity in *C. scolymus* to defined sesquiterpene lactones using a combined potency-abundance approach. Quantitative total bioactivity analysis (TBA) highlighted differences in the potency of each compound and their contributions to the overall DCM extract with respect to Nrf2 reporter induction. Aguerin B (**3**) exhibited the highest fold activity yet contributes marginally to total Nrf2 activity due to its low abundance in the DCM extract. Cynaropicrin (**2**), by contrast, combines adequate potency with the highest relative abundance, making it the dominant bioactive constituent at the extract level. This mismatch between the in vitro results and their relevance at the extract level has several practical implications. Cynaropicrin (**2**) is the appropriate marker compound for quality control and bioactivity-guided standardization of Nrf2-relevant globe artichoke preparations.

Comparative structure–activity analysis of the isolated compounds indicated that Nrf2-inducing potency was critically dependent on the presence of an intact electrophilic α-methylene-γ-lactone pharmacophore in conjunction with oxygenation at the C-8 position. Compounds retaining this structural feature exhibited robust Nrf2 reporter induction, whereas saturation of the exocyclic methylene and hydroxylation at C-8 in 8-deoxy-11,13-dihydroxygrosheimin (**5**) resulted in loss of activity. This finding aligns with established mechanistic models of canonical Nrf2 activation via covalent Michael addition, wherein nucleophilic thiols on highly sensitive cysteine sensors of Keap1 (such as Cys151) attack electron-deficient exocyclic methylene carbons. Although direct covalent adduct formation was not demonstrated here, it remains to be confirmed by targeted mass spectrometry or thiol-trapping approaches in future studies.

Functional redox-responsive readouts corroborated the reporter-based findings, indicating that upstream induction of the Nrf2/ARE pathway translated into measurable biochemical effects. Exposure of cells to the active sesquiterpene lactones elevated both extracellular GSH and cysteinyl-glycine (CysGly), consistent with Nrf2-driven upregulation of glutathione biosynthesis, subsequent GSH export, and γ-glutamyltransferase-mediated catabolism at the cell surface. The cytoprotection data against H_2_O_2_ provided the most functionally meaningful validation of this interpretation. If elevated thiol levels were a consequence of cytotoxicity rather than active biosynthesis, cytoprotection would not be observed. Since cellular H_2_O_2_ detoxification is mediated by glutathione peroxidase (GPx) and strictly depends on GSH, the concordance between compounds that elevated extracellular thiols and those that attenuated oxidative injury strengthens the case that these changes reflect a functional, Nrf2-dependent response.

The HO-1 induction by cynaropicrin (**2**) in HepG2 cells further confirmed that the reporter activity translates to protein-level downstream signaling. This indicates that activation of the reporter assay is not merely an isolated transcriptional readout but is accompanied by the upregulation of endogenous Nrf2 target genes.

Furthermore, the comparison of isolated compounds with clinically approved fumarates in the same AREc32 system is perhaps the most contextually significant finding from a translational perspective, though it requires careful framing. Both cynaropicrin (**2**) and aguerin B (**3**) outperformed the approved fumarates across the tested concentration range, with cynaropicrin (**2**) active at substantially lower concentrations. This is an in vitro observation in the AREc32 reporter cell line and does not account for the substantial pharmacokinetic differences between these compound classes.

Together, the reporter, HO-1, and glutathione data indicate that cynaropicrin (**2**) is the lead Nrf2-active constituent of globe artichoke and supports its prioritization for further mechanistic and in vivo studies.

The results are highly consistent with induction of the Nrf2/ARE pathway, although direct target engagement was not assessed, and Nrf2 nuclear translocation was not directly visualized in this study. Instead, pathway induction was inferred indirectly through ARE-luciferase reporter activity and downstream target gene/protein induction. Imaging-based confirmation of nuclear translocation remains an important next step for validating the proposed mechanism. Moreover, given the Michael-acceptor reactivity characteristic of these electrophilic sesquiterpene lactones, genotoxicity assessment (e.g., comet assay, micronucleus assay, or γH2AX foci quantification) would meaningfully strengthen their safety profile. This is particularly important because the same electrophilic properties that may enable sensor-cysteine engagement could also contribute to broader reactivity with cellular thiols or DNA at higher exposure levels. Therefore, these assays should be considered in future studies. Concurrently, evaluating the selectivity of cynaropicrin’s cysteine reactivity through competitive activity-based protein profiling or LC–MS/MS-based cysteine mapping would help determine whether it preferentially engages hyperreactive sensor cysteines, such as those in Keap1, or instead exhibits broader thiol reactivity, which may also have implications for off-target and DNA-associated effects at higher exposures. As an in vitro reporter-based study, these results should be regarded as a mechanistic and structural validation to support future studies into in vivo efficacy or clinical relevance. Subsequent mechanistic studies may be considered to address whether covalent Keap1 modification is the principal driver of Nrf2 activation and evaluate the compound’s activity and tolerability in neuronal and glial cellular models. Furthermore, future in vivo studies can include pharmacokinetic profiling, oral bioavailability, tolerability, target-tissue exposure, and pharmacodynamic readouts such as Nrf2 target-gene induction in relevant tissues.

Nonetheless, by establishing the chemical identity and functional redox outcomes of globe artichoke (*C. scolymus*) lactones, this work provides a valuable phytochemical framework necessary to guide future in vivo trials and targeted pharmacokinetic formulations.

## 5. Conclusions

In summary, this work clarifies the phytochemical basis of Nrf2-related bioactivity in globe artichoke and highlights the central role of specific guaianolide-type sesquiterpene lactones. Furthermore, these findings enable bioactivity-guided standardization of globe artichoke extracts and provide a framework for linking phytochemical composition to functional redox outcomes.

Together, the results contribute to a more precise understanding of Nrf2/ARE-associated redox signaling in globe artichoke-derived systems and establish a mechanistic foundation for exploring Nrf2/ARE-inducing sesquiterpene lactones as potential therapeutic leads for developing drugs targeting diseases, in which oxidative stress plays a central pathological role.

## Figures and Tables

**Table 1:** Nrf2-induced luciferase activity of the sequential extracts from globe artichoke.

Sequential Extracts	Nrf2/ARE Reporter Activity (Fold Induction Compared to Non-Activated Cells, Mean ± SD)	% Induction Compared to 50 μM tBHQ (Positive Control)
n-hexane	2.66 ± 2.01	17.40
DCM	54.40 ± 2.18	363.80
EtOAc	3.35 ± 2.33	21.47
EtOH	1.38 ± 0.38	6.63
MeOH	3.92 ± 2.92	18.84
Water	1.06 ± 0.26	7.06

Results represent mean ± SD of 3 individual experiments in triplicate.

**Table 2:** In vitro Nrf2 activity and yield of HPLC fractions of globe artichoke’s DCM extract.

GA Fraction Name	Active Concentration (μg/mL)	Nrf2/ARE Reporter Activity (Fold Induction Compared to Non-Activated Cells, Mean ± SD)	% Induction Compared to 50 μM tBHQ (Positive Control)
Fraction 1&2	100	0	0
Fraction 3	100	4.11 ± 3.54	34.2
Fraction 4	100	11.01 ± 2.12	82.2
Fraction 5	100	31.02 ± 14.11	231.5
Fraction 6	100	31.40 ± 14.29	234.3
Fraction 7	6.25	16.45 ± 5.18	127.6
Fraction 8	25	64.25 ± 7.08	465
Fraction 9	25	56.21 ± 8.3	407
Fraction 10	3.125	33.94 ± 7.48	299.3
Fraction 11	25	35.56 ± 11.60	277.8
Fraction 12	50	22.39 ± 10.15	173.5
Fraction 13	25	32.95 ± 10.34	279.2
Fraction 14	25	39.14 ± 11.13	362.2
Fraction 15	50	5.073 ± 4.07	45.9
Fraction 16	25	25.07 ± 3.46	181.6
Fraction 17	50	20.30 ± 6.53	157.3
Fraction 18	50	22.11 ± 6.57	171.4
Fraction 19	100	6.22 ± 2.37	48.2
Fraction 20	100	4.63 ± 1.97	35.8

Results represent mean ± SD of 3 individual experiments in triplicate.

**Table 3:** Total bioactivity (TBA) calculations for HPLC fractions of the GA DCM extract.

Fraction No.	Weight (mg)	Potency EC_50_ (μg/mL)	Potency EDV_50_ (L/g)	TBA (L^−1^) (EDV_50_ × Weight)	% TBA
F-1	0.6	55.1	18.15	0.01	0.11
F-2	0.8	66.13	15.12	0.01	0.12
F-3	4.6	166.3	6.01	0.03	0.29
F-4	10.2	394.1	2.54	0.03	0.27
F-5	7	55.65	17.97	0.13	1.30
F-6	15.4	221.6	4.51	0.07	0.72
F-7	11	10	100.00	1.10	11.34
F-8	7.6	46.86	21.34	0.16	1.67
F-9	12.8	26.3	38.02	0.49	5.02
F-10	14	2.293	436.11	6.11	62.92
F-11	10.2	54.83	18.24	0.19	1.92
F-12	7.8	19.12	52.30	0.41	4.20
F-13	3.4	21.25	47.06	0.16	1.65
F-14	2	13.55	73.80	0.15	1.52
F-15	1.9	30.59	32.69	0.06	0.64
F-16	4.8	13.35	74.91	0.36	3.71
F-17	3.6	31.06	32.20	0.12	1.19
F-18	6.2	106.5	9.39	0.06	0.60
F-19	20.5	432.9	2.31	0.05	0.49
F-20	23.5	713.9	1.40	0.03	0.34
Total for all fractions	167.9			9.70	100.00

Note: EDV_50_ values were calculated from EC_50_ values expressed in μg/mL by converting to g/L and taking the reciprocal. All measurements are based on the Nrf2 luciferase reporter assay.

## Data Availability

The original contributions presented in this study are included in the article. Further inquiries can be directed to the corresponding author.

## Supplementary Materials

The following supporting information can be downloaded at: https://www.mdpi.com/article/10.3390/biomedicines14071589/s1, Figure S1: Cell viability of sequential extracts of globe artichoke; Figure S2: Nrf2-inducing activity and cell viability of HPLC fractions derived from the DCM extract; Figure S3: HPLC-DAD chromatogram of GA_DCM fraction 6; Figure S4: LC–MS/PDA trace of isolated sesquiterpene lactones; Figure S5: ^1^H NMR spectrum of grosheimin 1 (400 MHz, MeOH-d4); Figure S6: ^1^H NMR spectrum of cynaropicrin 2 (400 MHz, DMSO-d6); Figure S7: ^1^H NMR spectrum of aguerin B 3 (600 MHz, DMSO-d6); Figure S8: ^1^H NMR spectrum of janerin 4 (600 MHz, DMSO-d6); Figure S9: ^1^H NMR spectrum of 8-deoxy-11,13-dihydroxygrosheimin 5 (600 MHz, DMSO-d6); Figure S10: HRMS (ESI+) spectrum of grosheimin 1; Figure S11: HRMS (ESI+) spectrum of cynaropicrin 2; Figure S12: HRMS (ESI+) spectrum of aguerin B 3; Figure S13: HRMS (ESI+) spectrum of janerin 4; Figure S14: HRMS (ESI+) spectrum of 8-deoxy-11,13-dihydroxygrosheimin 5; Figure S15: Cynaropicrin induces HO-1 protein expression in HepG2 cells.

## Abbreviations

The following abbreviations are used in this manuscript:

Keap1	Kelch-like ECH-associated protein1
Nrf2	nuclear factor erythroid 2–related factor 2
ARE	antioxidant response element
DCM	dichloromethane
GAPDH	Glyceraldehyde 3-phosphate dehydrogenase
HO-1	heme oxygenase-1
GSH	glutathione
CysGly	cysteinylglycine
NQO1	NAD(P)H:quinone oxidoreductase 1
GST	glutathione S-transferase
RLU	relative luminescence units
ROS	reactive oxygen species
tBHQ	tert-butylhydroquinone
H_2_O_2_	hydrogen peroxide
