# Supplementary material for: Guaianolide Sesquiterpene Lactones from Globe Artichoke (Cynara scolymus L.) Induce Nrf2-Associated Antioxidant Signaling
Source: Biomedicines. 2026 Jul 16;14(7):1589. doi: 10.3390/biomedicines14071589 (PMC13406214; doi:10.3390/biomedicines14071589)
Supplement: Supplementary file 1 [file biomedicines-14-01589-s001.zip › biomedicines-4380088-supplementary.pdf]

Supplementary Information

Guaianolide sesquiterpene lactones from globe artichoke (*Cynara scolymus* L.) induce Nrf2-associated antioxidant signaling

Preeti Kushwaha[1], Sualiha Afzal[1], Ritesh Raju[1], Xian Zhou[2], Gerald Münch[1,2]*

[1] Pharmacology Unit, Western Sydney University, Campbelltown, NSW 2560, Australia
[2] NICM Health Research Institute, Western Sydney University, Westmead, NSW 2145, Australia
* Correspondence: g.muench@westernsydney.edu.au; Tel.: +61 2 46203814

Supplementary data provides additional biological, chromatographic and spectroscopic validation supporting the identification and functional characterisation of Nrf2-active constituents described in the main text

Table of Contents

Figure S1: Cell viability of sequential extracts of globe artichoke. 3

Figure S2: Nrf2-inducing activity and cell viability of HPLC fractions derived from the DCM extract. 10

Figure S3: HPLC-DAD chromatogram of GA_DCM fraction 6 11

Figure S4: LC–MS/PDA trace of isolated sesquiterpene lactones. 12

Figure S5: ^{1}H NMR spectrum of grosheimin 1 (400 MHz, MeOH-d_4) 13

Figure S6: ^{1}H NMR spectrum of cynaropicrin 2 (400 MHz, DMSO-d_6) 14

Figure S7: ^{1}H NMR spectrum of aguerin B 3 (600 MHz, DMSO-d_6) 15

Figure S8: ^{1}H NMR spectrum of janerin 4 (600 MHz, DMSO-d_6) 16

Figure S9: ^{1}H NMR spectrum of 8-deoxy-11,13-dihydroxygrosheimin 5 (600 MHz, DMSO-d_6) 17

Figure S10: HRMS (ESI$^+$) spectrum of grosheimin 1 18

Figure S11: HRMS (ESI$^+$) spectrum of cynaropicrin 2 19

Figure S12: HRMS (ESI$^+$) spectrum of aguerin B 3 20

Figure S13: HRMS (ESI$^+$) spectrum of janerin 4 21

Figure S14: HRMS (ESI$^+$) spectrum of 8-deoxy-11,13-dihydroxygrosheimin 5 22

Figure S15: Cynaropicrin induces HO-1 protein expression in HepG2 cells 23

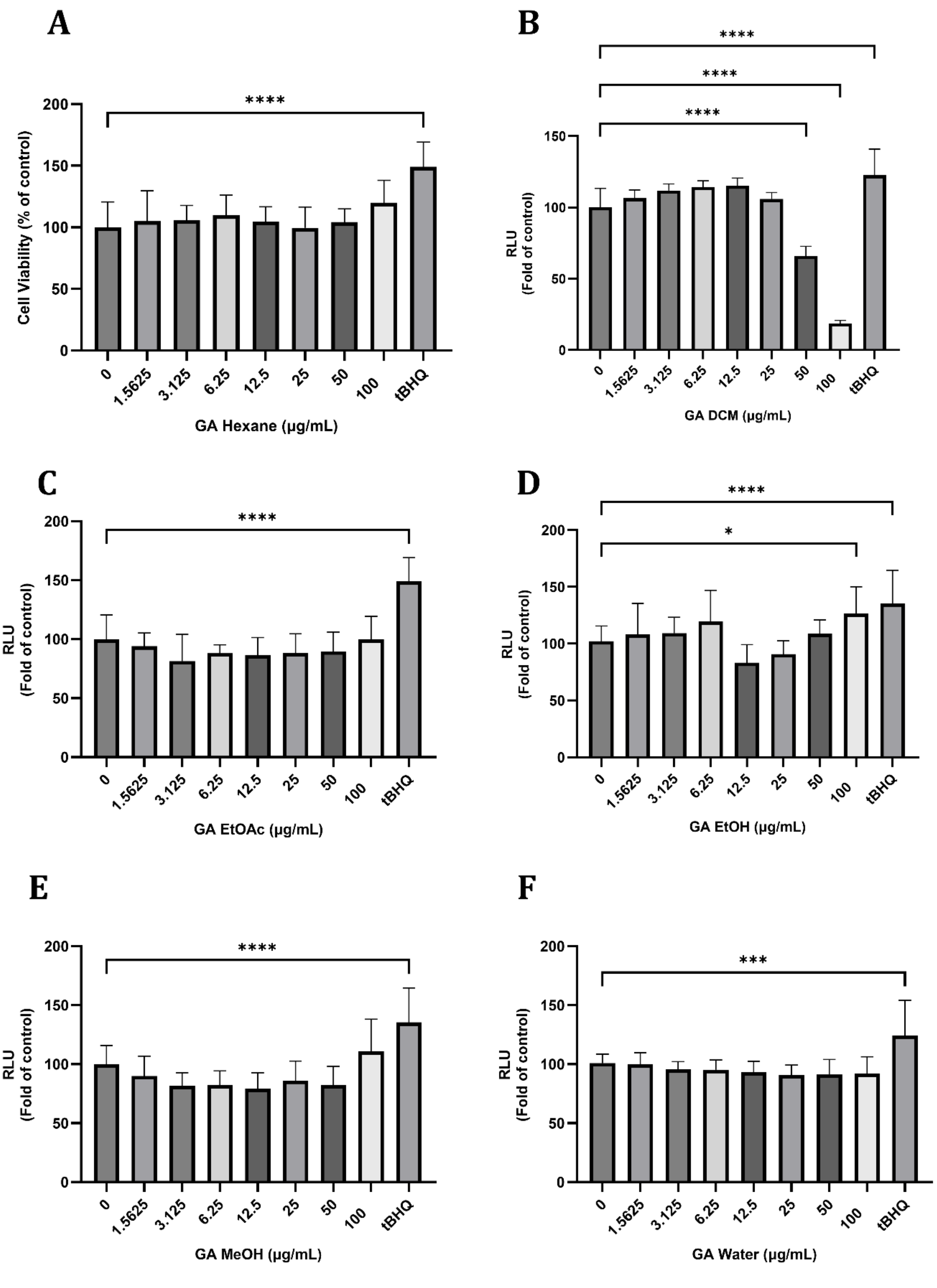

Figure S1: Cell viability of sequential extracts of globe artichoke.

AREc32 cells were treated with serial dilutions of each solvent extract (µg/mL) and tBHQ (50 µM) for 24 h. Data represent the mean ± SD of 3 individual experiments in triplicate. Significance was assessed by one-way ANOVA, ****$p < 0.0001$ *vs.* control (concentration = 0).. Panels: A) n-hexane, B) DCM, C) EtOAc, D) EtOH, E) MeOH, F) water.

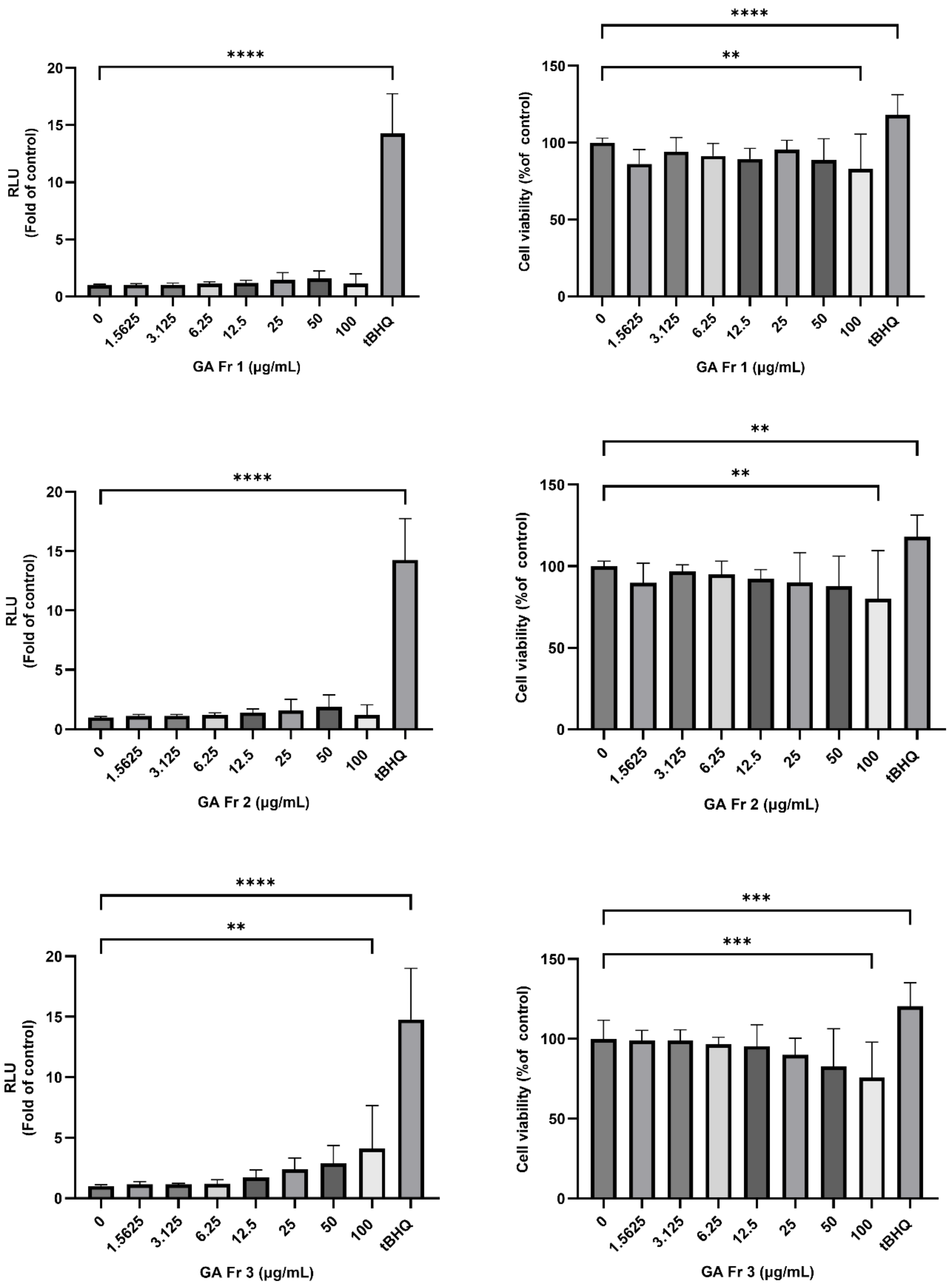
****
RLU
(Fold of control)
20
15
10
5
0
0
1.5625
3.125
6.25
12.5
25
50
100
tBHQ
GA Fr 1 (µg/mL)
****
**
Cell viability (%of control)
150
100
50
0
0
1.5625
3.125
6.25
12.5
25
50
100
tBHQ
GA Fr 1 (µg/mL)
****
RLU
(Fold of control)
20
15
10
5
0
0
1.5625
3.125
6.25
12.5
25
50
100
tBHQ
GA Fr 2 (µg/mL)
**
**
Cell viability (%of control)
150
100
50
0
0
1.5625
3.125
6.25
12.5
25
50
100
tBHQ
GA Fr 2 (µg/mL)
****
**
RLU
(Fold of control)
20
15
10
5
0
0
1.5625
3.125
6.25
12.5
25
50
100
tBHQ
GA Fr 3 (µg/mL)
***
***
Cell viability (%of control)
150
100
50
0
0
1.5625
3.125
6.25
12.5
25
50
100
tBHQ
GA Fr 3 (µg/mL)

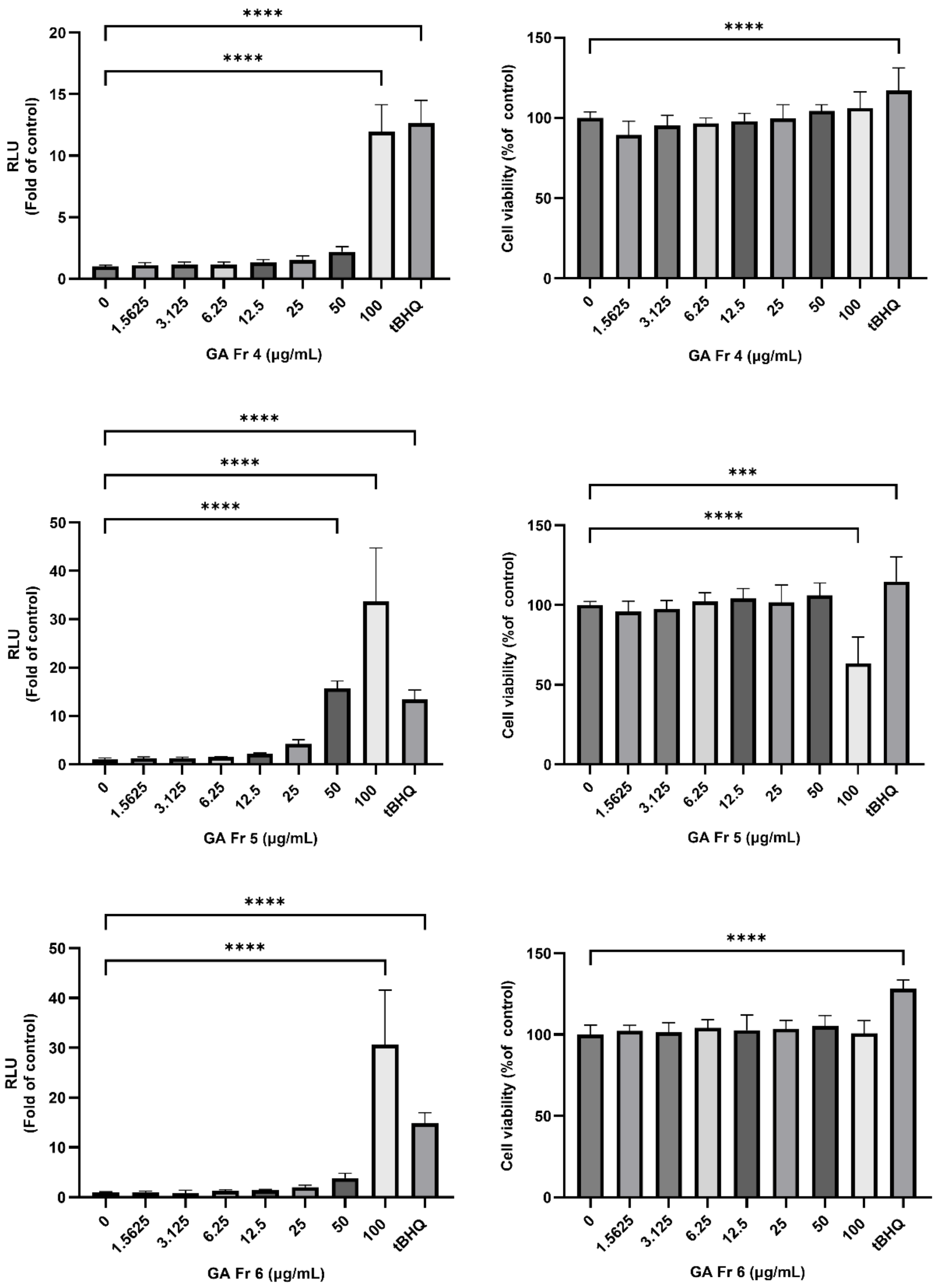

RLU
(Fold of control)
GA Fr 4 (µg/mL)
Cell viability (%of control)
GA Fr 4 (µg/mL)
RLU
(Fold of control)
GA Fr 5 (µg/mL)
Cell viability (%of control)
GA Fr 5 (µg/mL)
RLU
(Fold of control)
GA Fr 6 (µg/mL)
Cell viability (%of control)
GA Fr 6 (µg/mL)
0
1.5625
3.125
6.25
12.5
25
50
100
tBHQ
****
***

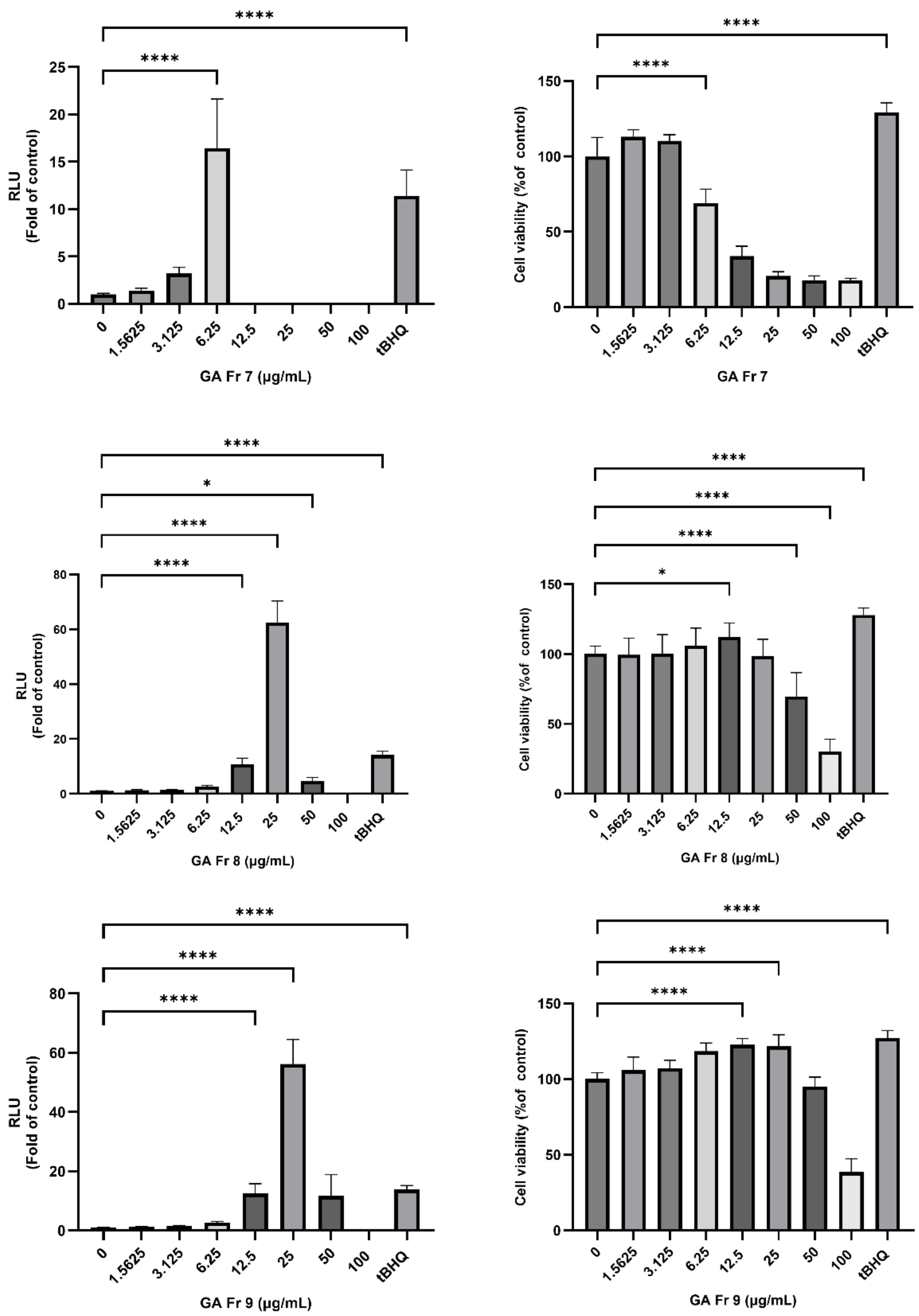
****
****
RLU
(Fold of control)
25
20
15
10
5
0
0
1.5625
3.125
6.25
12.5
25
50
100
tBHQ
GA Fr 7 (μg/mL)
****
****
Cell viability (%of control)
150
100
50
0
0
1.5625
3.125
6.25
12.5
25
50
100
tBHQ
GA Fr 7
****
*
****
****
RLU
(Fold of control)
80
60
40
20
0
0
1.5625
3.125
6.25
12.5
25
50
100
tBHQ
GA Fr 8 (μg/mL)
****
****
****
*
Cell viability (%of control)
150
100
50
0
0
1.5625
3.125
6.25
12.5
25
50
100
tBHQ
GA Fr 8 (μg/mL)
****
****
****
RLU
(Fold of control)
80
60
40
20
0
0
1.5625
3.125
6.25
12.5
25
50
100
tBHQ
GA Fr 9 (μg/mL)
****
****
****
Cell viability (%of control)
150
100
50
0
0
1.5625
3.125
6.25
12.5
25
50
100
tBHQ
GA Fr 9 (μg/mL)

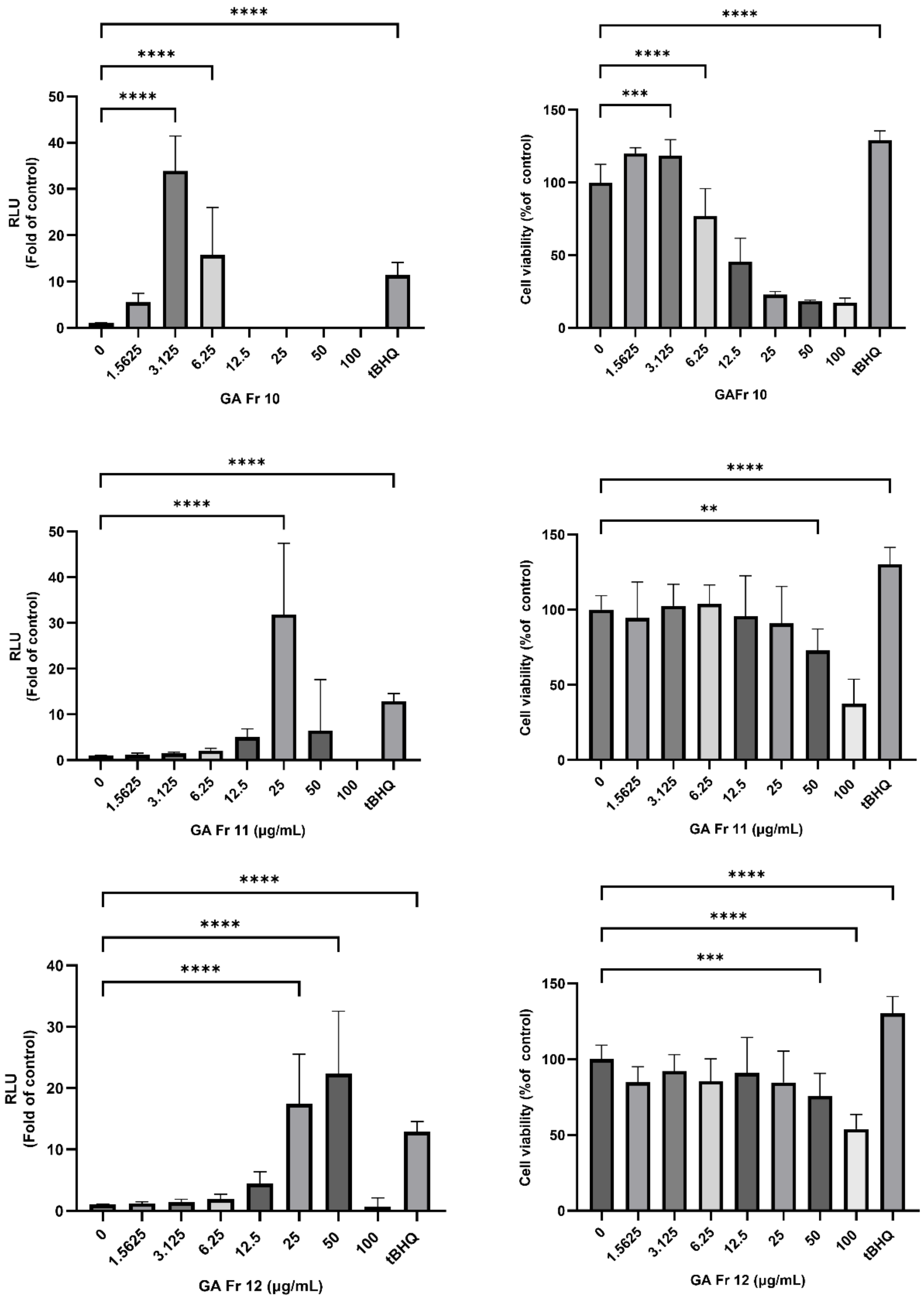
****
****
****
RLU
(Fold of control)
50
40
30
20
10
0
0
1.5625
3.125
6.25
12.5
25
50
100
tBHQ
GA Fr 10
****
****
***
Cell viability (%of control)
150
100
50
0
GAFr 10
****
****
RLU
(Fold of control)
GA Fr 11 (μg/mL)
****
**
Cell viability (%of control)
GA Fr 11 (μg/mL)
****
****
****
RLU
(Fold of control)
40
30
20
10
0
GA Fr 12 (μg/mL)
****
****
***
Cell viability (%of control)
GA Fr 12 (μg/mL)

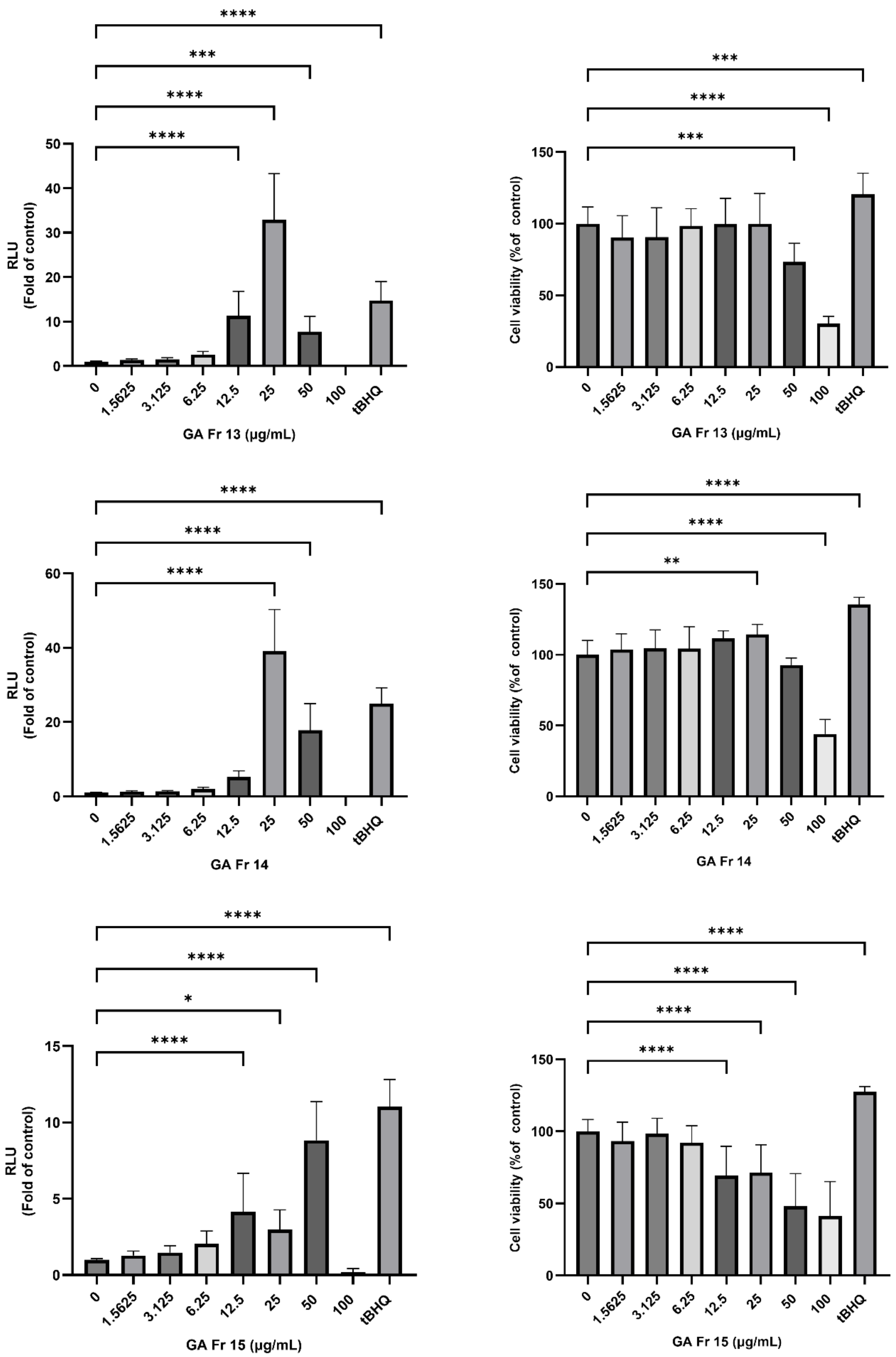
****
***
****
****
RLU
(Fold of control)
0
10
20
30
40
50
0
1.5625
3.125
6.25
12.5
25
50
100
tBHQ
GA Fr 13 (µg/mL)
***
****
***
Cell viability (%of control)
0
50
100
150
0
1.5625
3.125
6.25
12.5
25
50
100
tBHQ
GA Fr 13 (µg/mL)
****
****
****
RLU
(Fold of control)
0
20
40
60
0
1.5625
3.125
6.25
12.5
25
50
100
tBHQ
GA Fr 14
****
****
**
Cell viability (%of control)
0
50
100
150
0
1.5625
3.125
6.25
12.5
25
50
100
tBHQ
GA Fr 14
****
****
*
****
RLU
(Fold of control)
0
5
10
15
0
1.5625
3.125
6.25
12.5
25
50
100
tBHQ
GA Fr 15 (µg/mL)
****
****
****
****
Cell viability (%of control)
0
50
100
150
0
1.5625
3.125
6.25
12.5
25
50
100
tBHQ
GA Fr 15 (µg/mL)

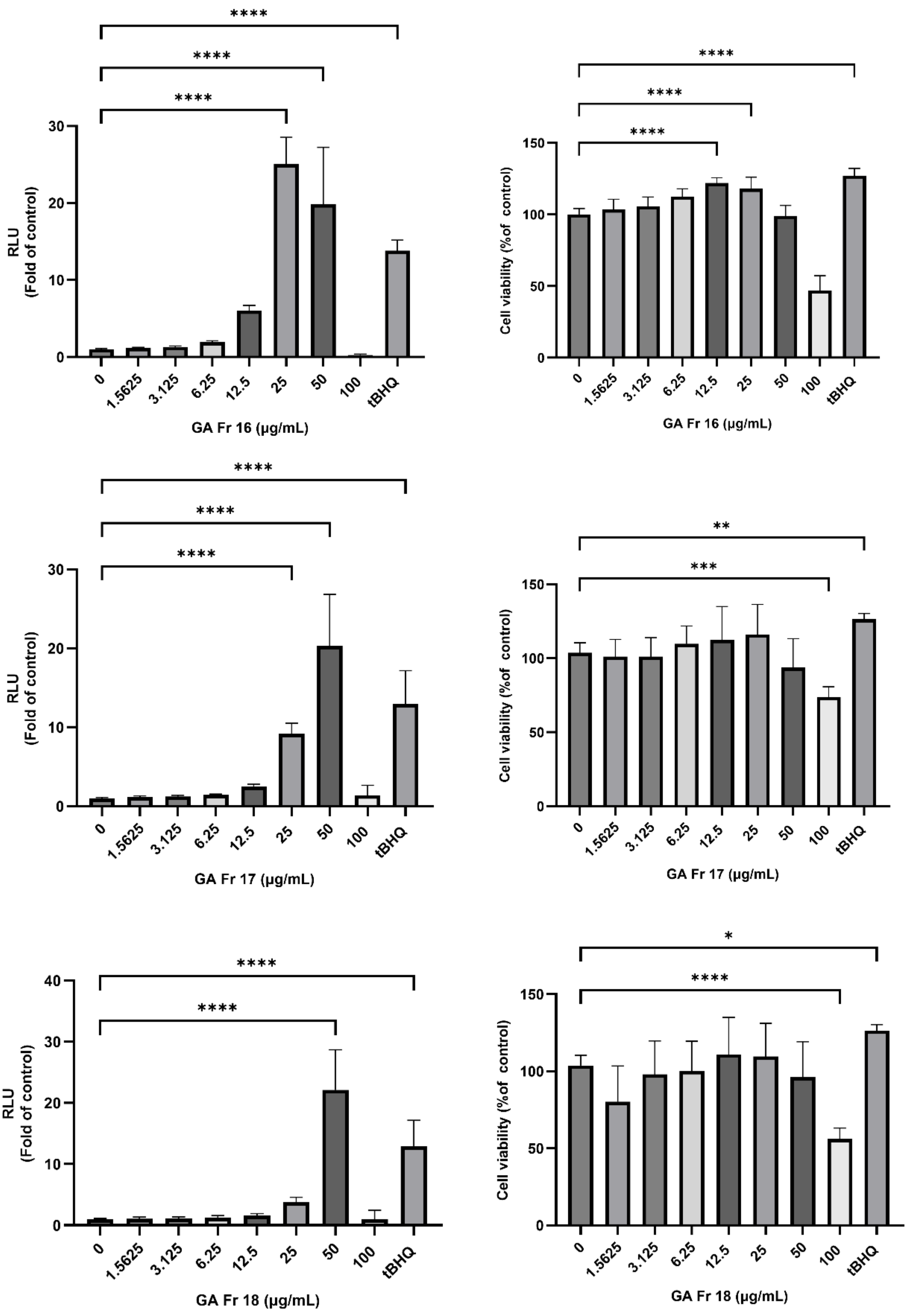
****
****
****
RLU
(Fold of control)
0
10
20
30
0
1.5625
3.125
6.25
12.5
25
50
100
tBHQ
GA Fr 16 (µg/mL)
****
****
****
Cell viability (%of control)
0
50
100
150
0
1.5625
3.125
6.25
12.5
25
50
100
tBHQ
GA Fr 16 (µg/mL)
****
****
****
RLU
(Fold of control)
0
10
20
30
0
1.5625
3.125
6.25
12.5
25
50
100
tBHQ
GA Fr 17 (µg/mL)
**
***
Cell viability (%of control)
0
50
100
150
0
1.5625
3.125
6.25
12.5
25
50
100
tBHQ
GA Fr 17 (µg/mL)
****
****
RLU
(Fold of control)
0
10
20
30
40
0
1.5625
3.125
6.25
12.5
25
50
100
tBHQ
GA Fr 18 (µg/mL)
*
****
Cell viability (%of control)
0
50
100
150
0
1.5625
3.125
6.25
12.5
25
50
100
tBHQ
GA Fr 18 (µg/mL)

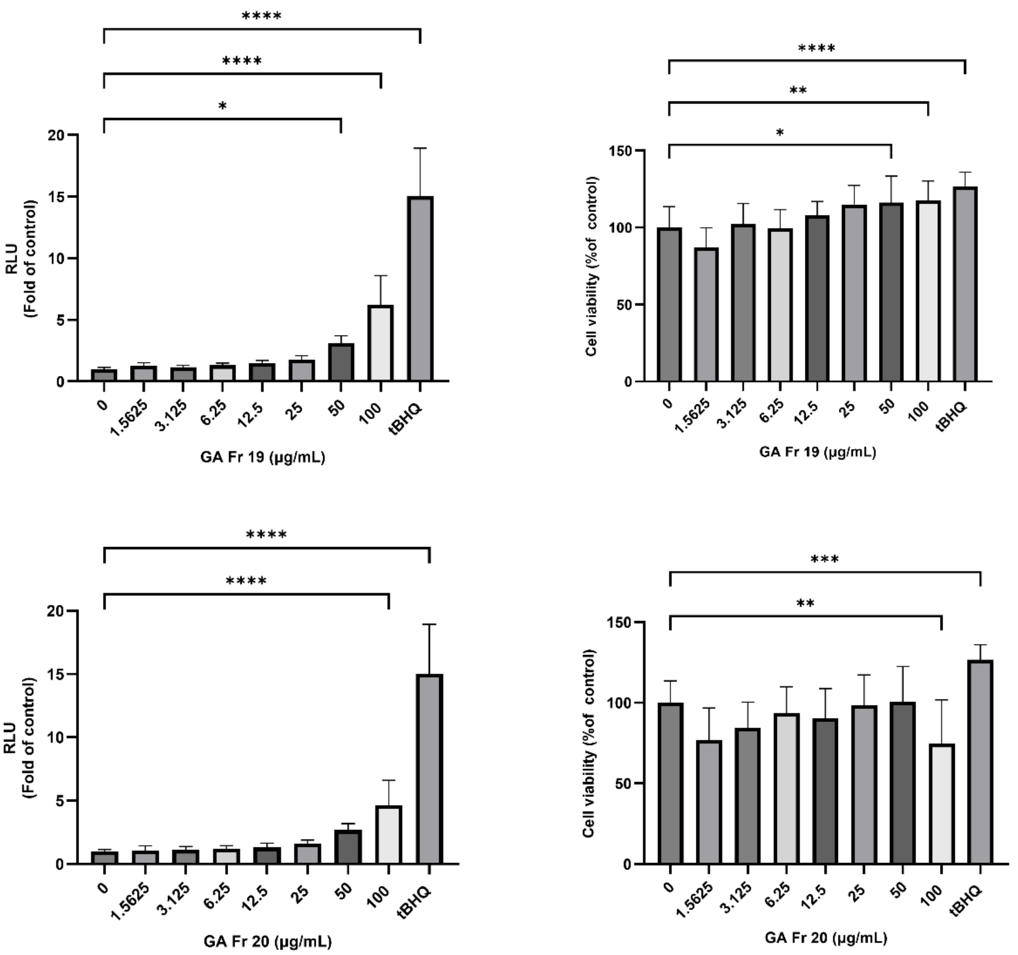

Figure S2: Nrf2-inducing activity and cell viability of HPLC fractions derived from the DCM extract.
AREc32 cells were exposed to serial dilutions of HPLC fractions (1–20) obtained from the dichloromethane extract. Nrf2 activity was quantified using a luciferase reporter assay, and cell viability was assessed in parallel using Alamar Blue. Data represent the mean ± SD of 3 individual experiments in triplicate. Significance was compared by one-way ANOVA analysis; *p<0.05, ** $p < 0.01$, *** $p < 0.001$, ****$p < 0.0001$ *vs.* control (concentration = 0).

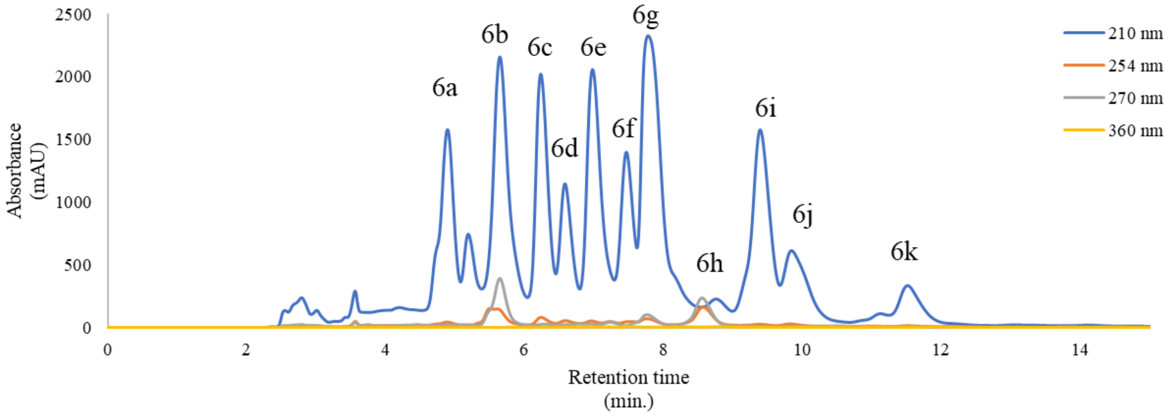

Figure S3: HPLC-DAD chromatogram of GA_DCM fraction 6.

Chromatographic profile of fraction 6 obtained from the DCM extract, used for secondary purification. Separation was performed on an XDB-C18 column (4.6 × 250 mm, 5 µm) using isocratic elution (25% MeCN/H_2O, 0.01% formic acid) at 0.8 mL/min. The chromatogram shows discrete peaks suitable for further isolation of individual compounds, including janerin and related constituents as described in the main text.

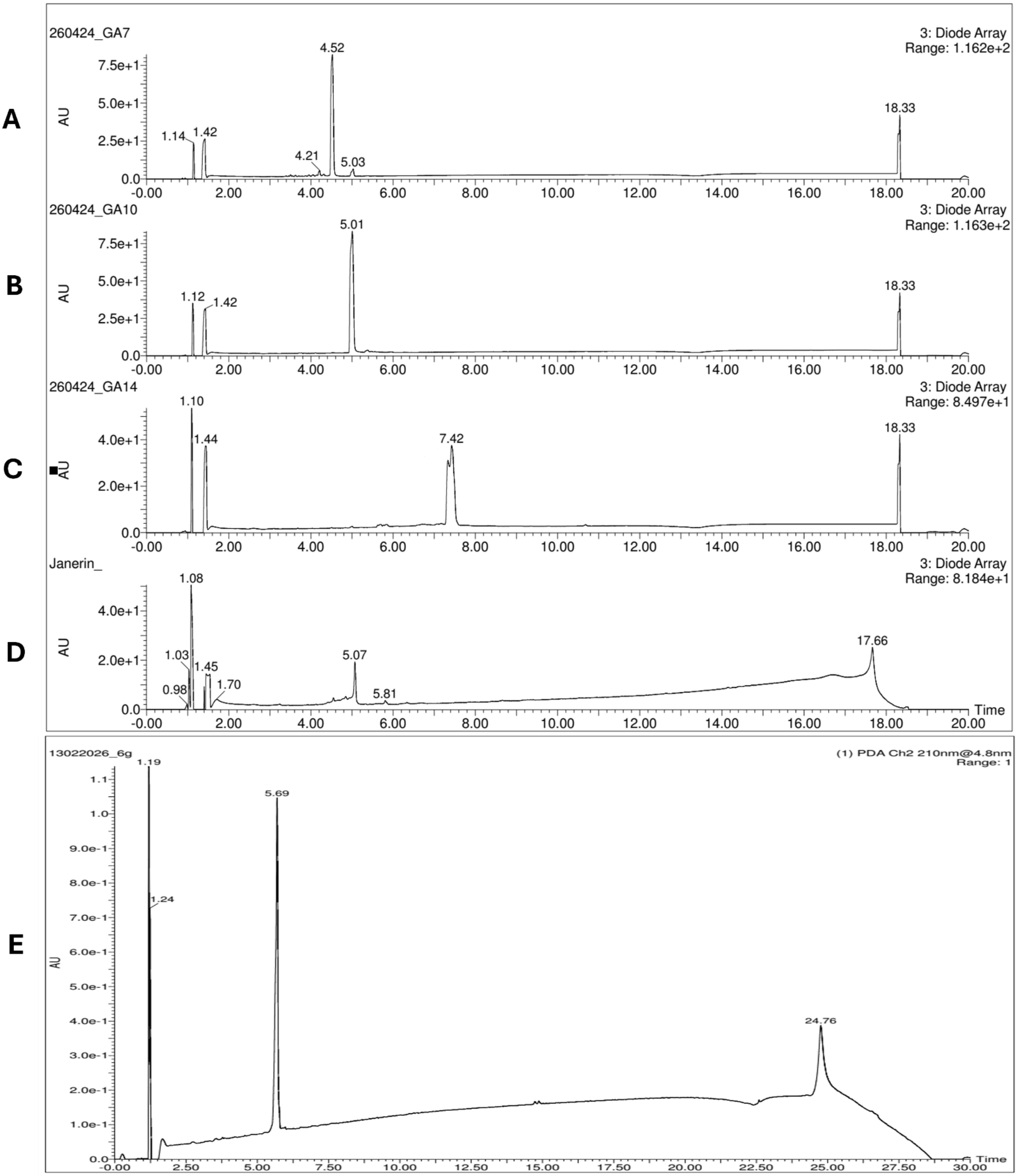

Figure S4: LC–MS/PDA trace of isolated sesquiterpene lactones.

LCMS was carried out on a Waters Aquity uUPLC-TQ MS with a PDA detector in positive ESI and negative ESI mode. A varying gradient of MeCN and H_2O (with 0.1% FA) was used as solvent system, with a flow rate of 0.3 mL/min on an Acquity uUPLC BEH C18 1.7 µM, 2.1 x 150 mm column was used. The UV detection wavelengths were set between 190-400 nm. Panel: **A**) grosheimin, **B**) cynaropicrin, **C**) aguerin B, **D**) janerin, and **E**) 8-deoxy-11,13-dihydroxygrosheimin

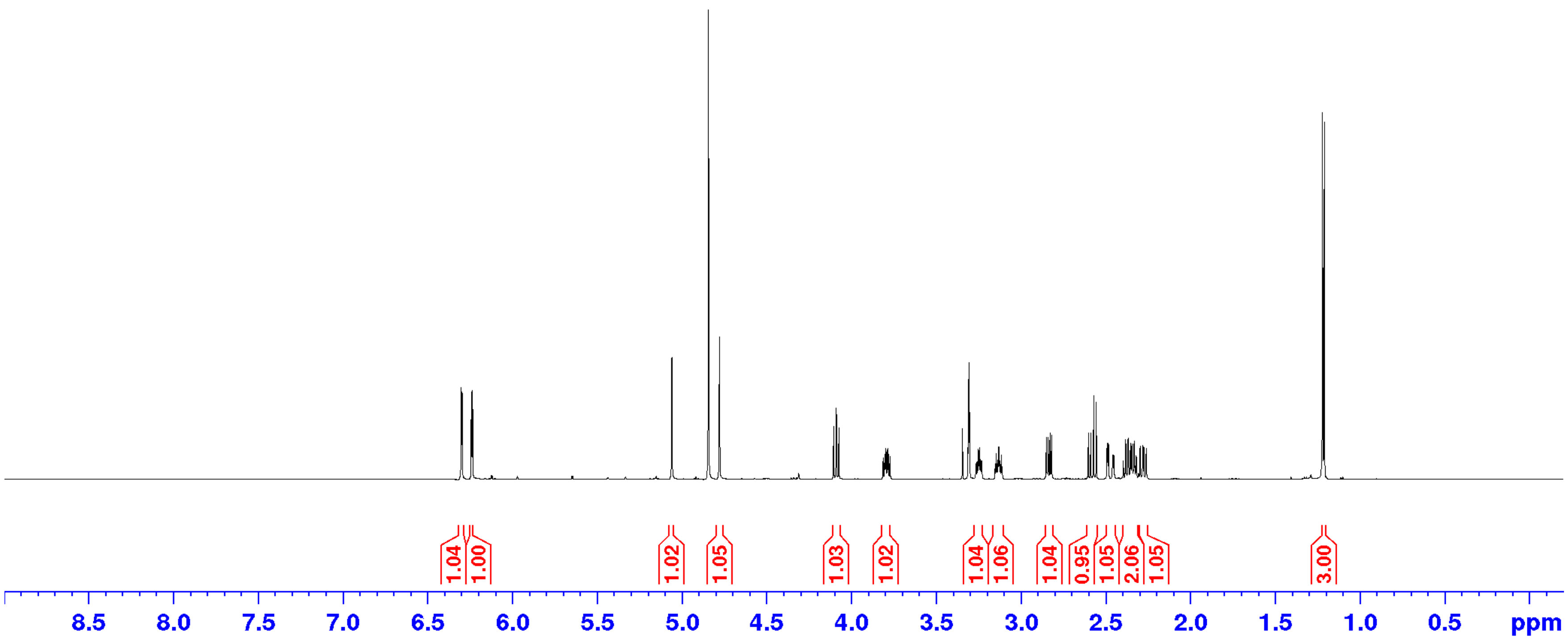

Figure S5: ^{1}H NMR spectrum of grosheimin 1 (400 MHz, MeOH-d_4)

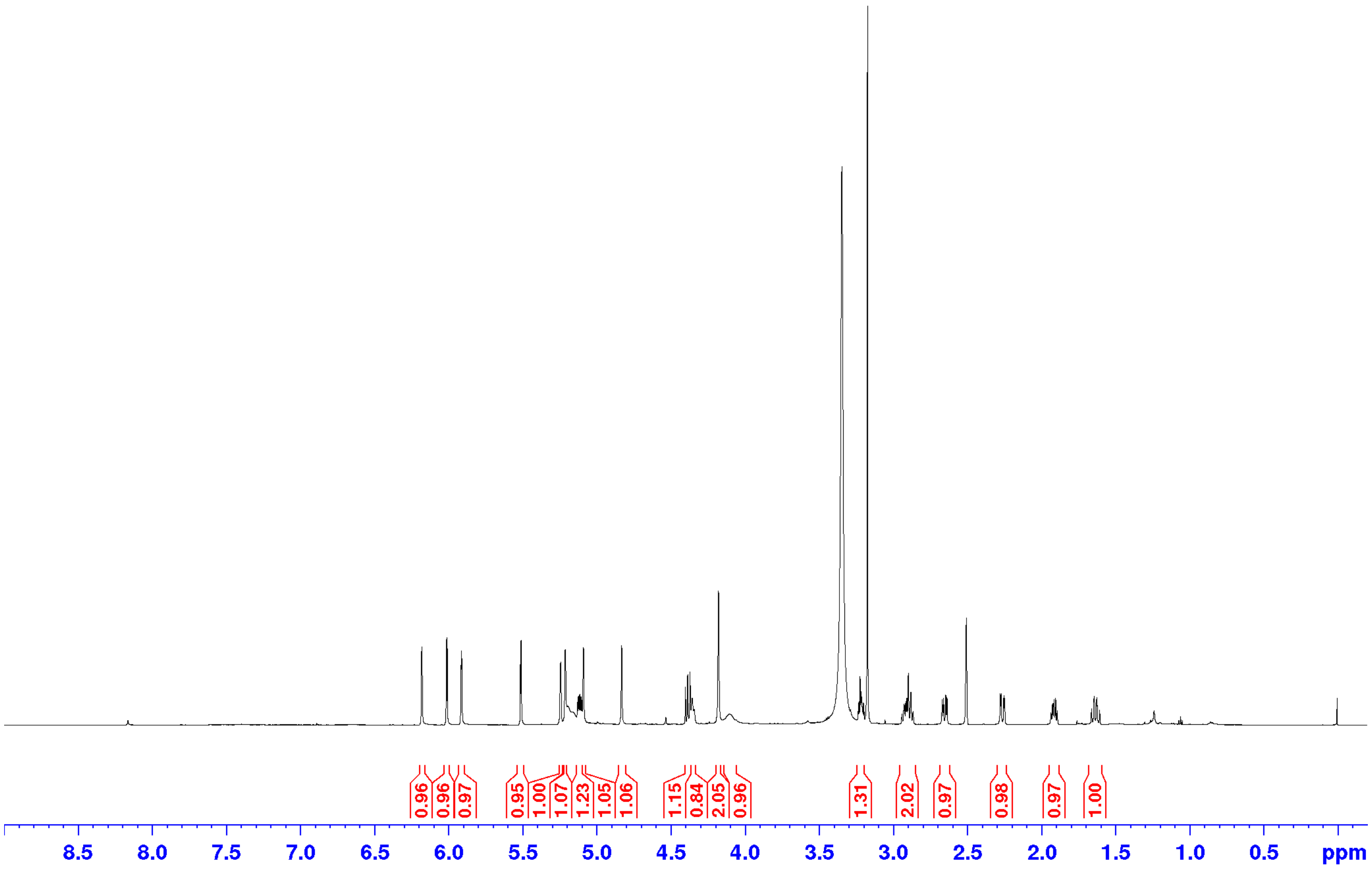

Figure S6: ^{1}H NMR spectrum of cynaropicrin 2 (400 MHz, DMSO-*d*$_6$)

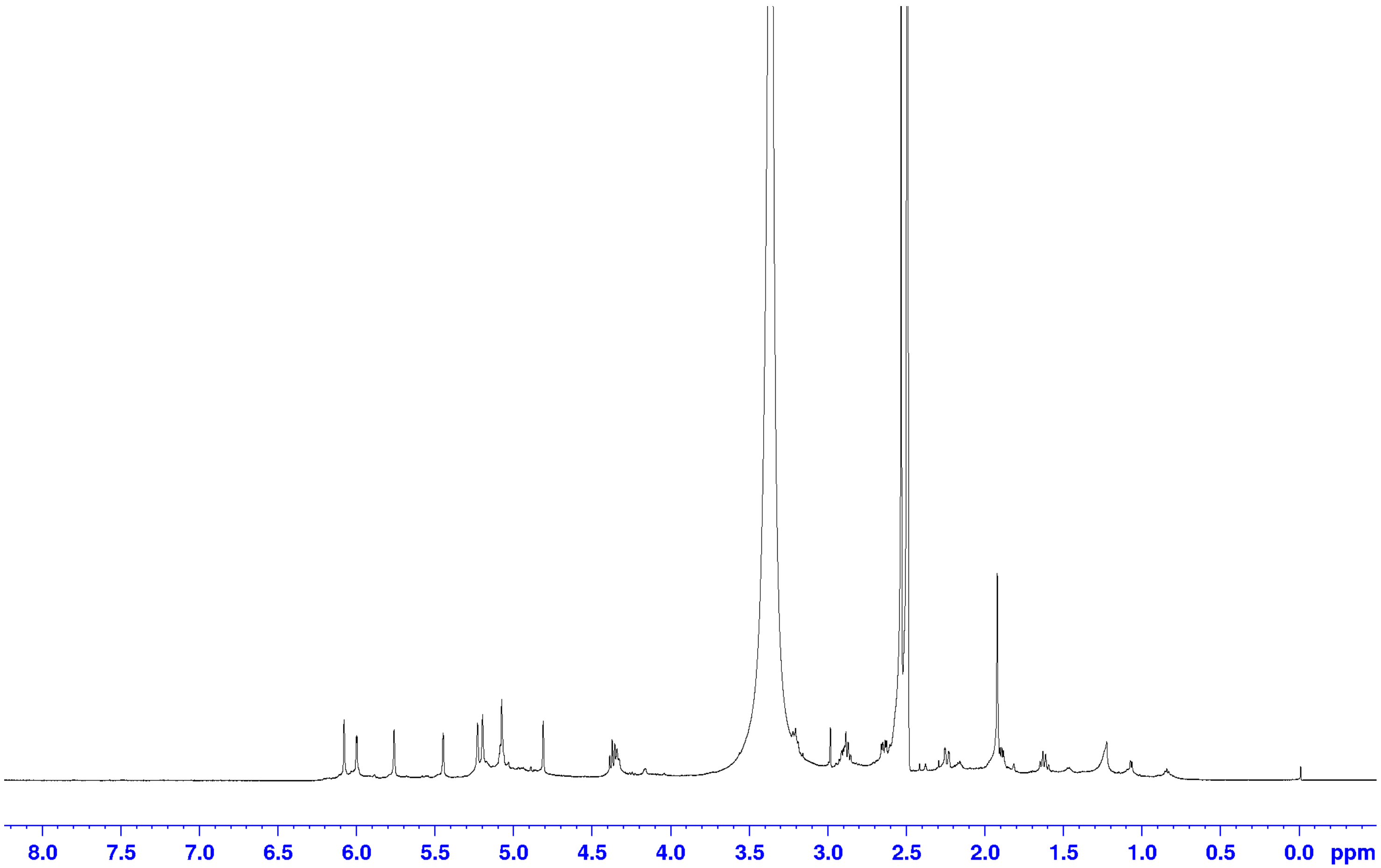

Figure S7: ^{1}H NMR spectrum of aguerin B 3 (600 MHz, DMSO-d_6)

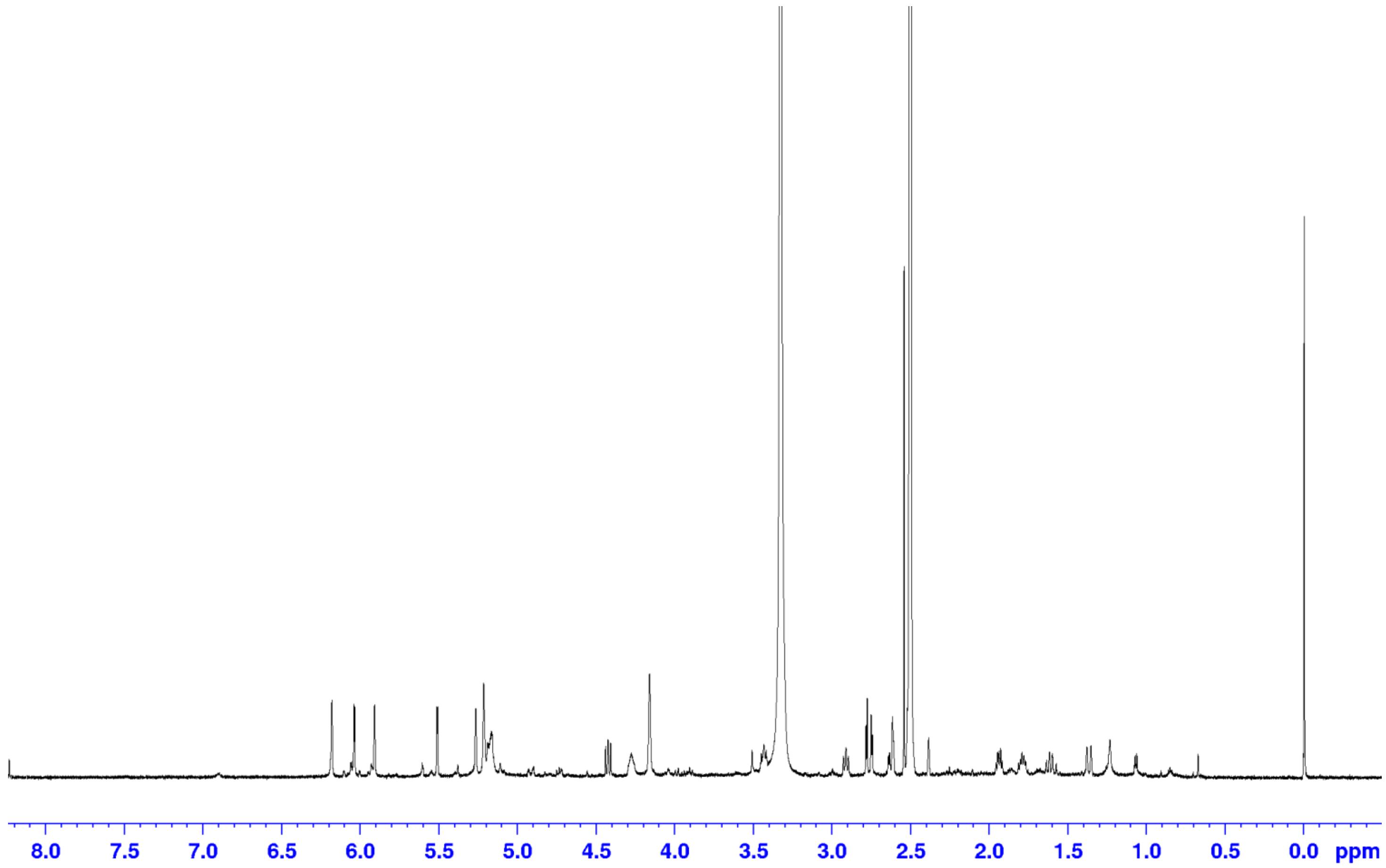

Figure S8: ^{1}H NMR spectrum of janerin 4 (600 MHz, DMSO-d_6)

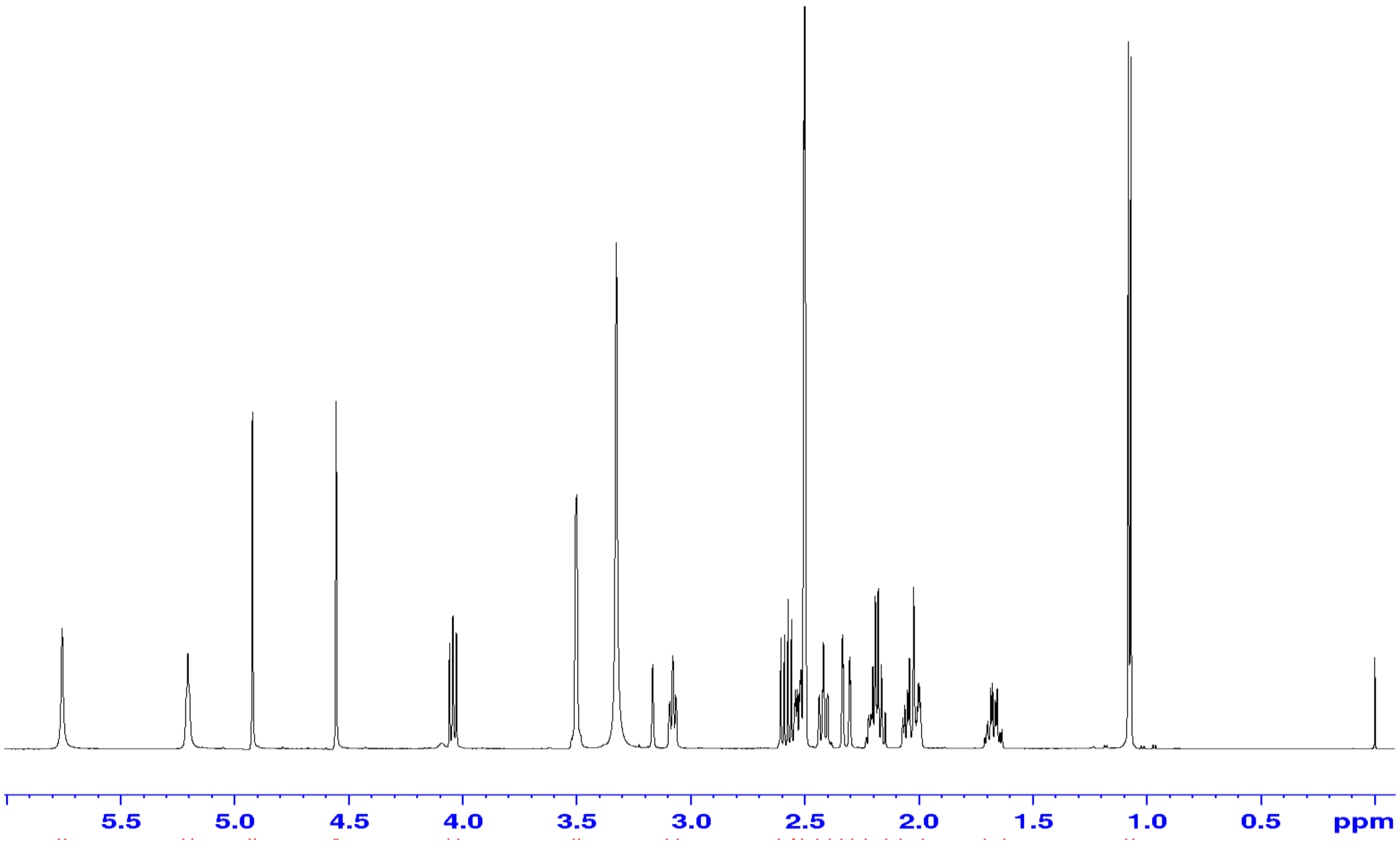

Figure S9: ^{1}H NMR spectrum of 8-deoxy-11,13-dihydroxygrosheimin 5 (600 MHz, DMSO-*d_6*)

Elemental Composition Report

Page 1

Single Mass Analysis

Tolerance = 100.0 PPM / DBE: min = -1.5, max = 50.0
Element prediction: Off
Number of isotope peaks used for i-FIT = 3

Monoisotopic Mass, Even Electron Ions
12 formula(e) evaluated with 1 results within limits (up to 50 closest results for each mass)
Elements Used:
C: 0-15 H: 0-20 O: 0-6 23Na: 0-1
7
24102025_GA_7 68 (1.345) AM2 (Ar,20000.0,0.00,0.00); ABS; Cm (23:71)

1: TOF MS ES+
2.29e+006

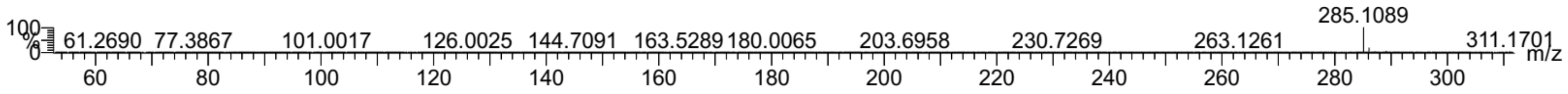

	Mass	Calc. Mass	mDa	PPM	DBE	i-FIT	Norm	Conf(%)	Formula
Minimum:					-1.5				
Maximum:			250.0	100.0	50.0				
	285.1089	285.1103	-1.4	-4.9	6.5	755.9	n/a	n/a	C15 H18 O4 23Na

Figure S10: HRMS (ESI[+]) spectrum of grosheimin 1

Single Mass Analysis

Tolerance = 100.0 PPM / DBE: min = -1.5, max = 50.0
Element prediction: Off
Number of isotope peaks used for i-FIT = 3

Monoisotopic Mass, Even Electron Ions
14 formula(e) evaluated with 1 results within limits (up to 50 closest results for each mass)
Elements Used:
C: 0-19 H: 0-22 O: 0-7 23Na: 0-1

346
GA_Fr_10 287 (5.581) AM2 (Ar,20000.0,556.28,0.00,LS 1); ABS

1: TOF MS ES+
2.72e+006

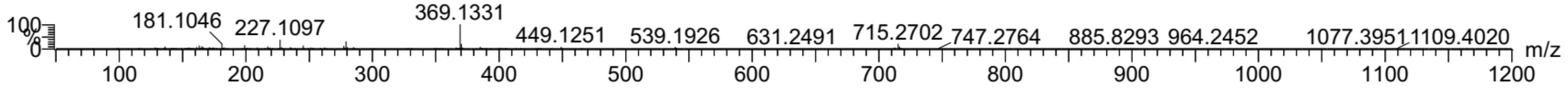

Minimum:				-1.5				
Maximum:		250.0	100.0	50.0				
Mass	Calc. Mass	mDa	PPM	DBE	i-FIT	Norm	Conf(%)	Formula
369.1331	369.1314	1.7	4.6	8.5	478.2	n/a	n/a	C19 H22 O6 23Na

Figure S11: HRMS (ESI+) spectrum of cynaropicrin 2

Elemental Composition Report

Single Mass Analysis
Tolerance = 100.0 PPM / DBE: min = -1.5, max = 50.0
Element prediction: Off
Number of isotope peaks used for i-FIT = 3

Monoisotopic Mass, Even Electron Ions
15 formula(e) evaluated with 1 results within limits (up to 50 closest results for each mass)
Elements Used:
C: 0-19 H: 0-22 O: 0-7 23Na: 0-1
GA_Fr_14 149 (2.912) AM2 (Ar,20000.0,556.28,0.00,LS 1); ABS
1: TOF MS ES+

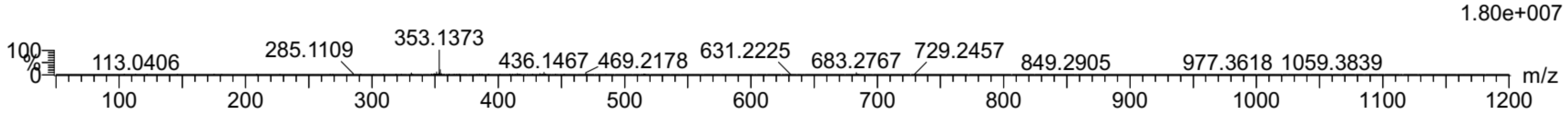

Minimum:				-1.5				
Maximum:		250.0	100.0	50.0				
Mass	Calc. Mass	mDa	PPM	DBE	i-FIT	Norm	Conf(%)	Formula
353.1373	353.1365	0.8	2.3	8.5	627.0	n/a	n/a	C19 H22 O5 23Na

Figure S12: HRMS (ESI+) spectrum of aguerin B 3

Elemental Composition Report

Single Mass Analysis

Tolerance = 100.0 PPM / DBE: min = -1.5, max = 50.0
Element prediction: Off
Number of isotope peaks used for i-FIT = 3

Monoisotopic Mass, Even Electron Ions
14 formula(e) evaluated with 1 results within limits (up to 50 closest results for each mass)
Elements Used:
C: 0-19 H: 0-22 O: 0-7 23Na: 0-1
7
24102025_GA_6E 92 (1.810) AM2 (Ar,20000.0,0.00,0.00); ABS; Cm (24:145) 1: TOF MS ES+
9.19e+006

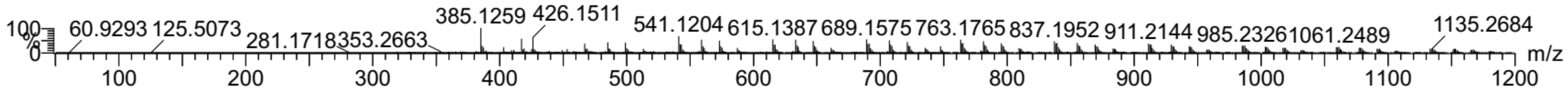

Mass	Calc. Mass	mDa	PPM	DBE	i-FIT	Norm	Conf(%)	Formula
Minimum:				-1.5				
Maximum:		250.0	100.0	50.0				
385.1259	385.1263	-0.4	-1.0	8.5	538.0	n/a	n/a	C19 H22 O7 23Na

Figure S13: HRMS (ESI⁺) spectrum of janerin 4

Elemental Composition Report

Single Mass Analysis

Tolerance = 100.0 PPM / DBE: min = -1.5, max = 50.0
Element prediction: Off
Number of isotope peaks used for i-FIT = 3

Monoisotopic Mass, Even Electron Ions
13 formula(e) evaluated with 1 results within limits (up to 50 closest results for each mass)
Elements Used:
C: 0-15 H: 0-20 O: 0-6 23Na: 0-1
6g
251022_GA_6g 148 (2.895) AM2 (Ar,20000.0,0.00,0.00); ABS; Cm (20:158)

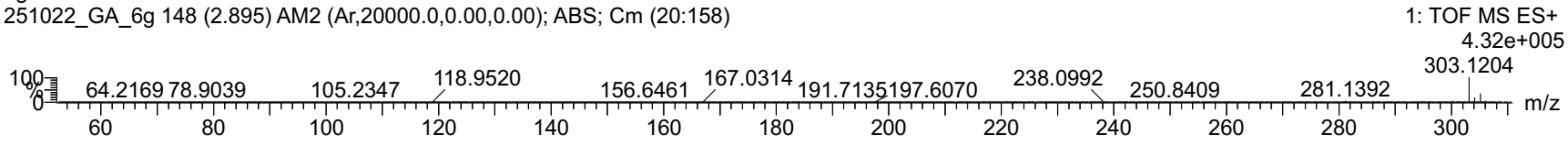

Minimum:				-1.5				
Maximum:		250.0	100.0	50.0				
Mass	Calc. Mass	mDa	PPM	DBE	i-FIT	Norm	Conf(%)	Formula
303.1204	303.1208	-0.4	-1.3	5.5	611.2	n/a	n/a	C15 H20 O5 23Na

Figure S14: HRMS (ESI+) spectrum of 8-deoxy-11,13-dihydroxygrosheimin 5

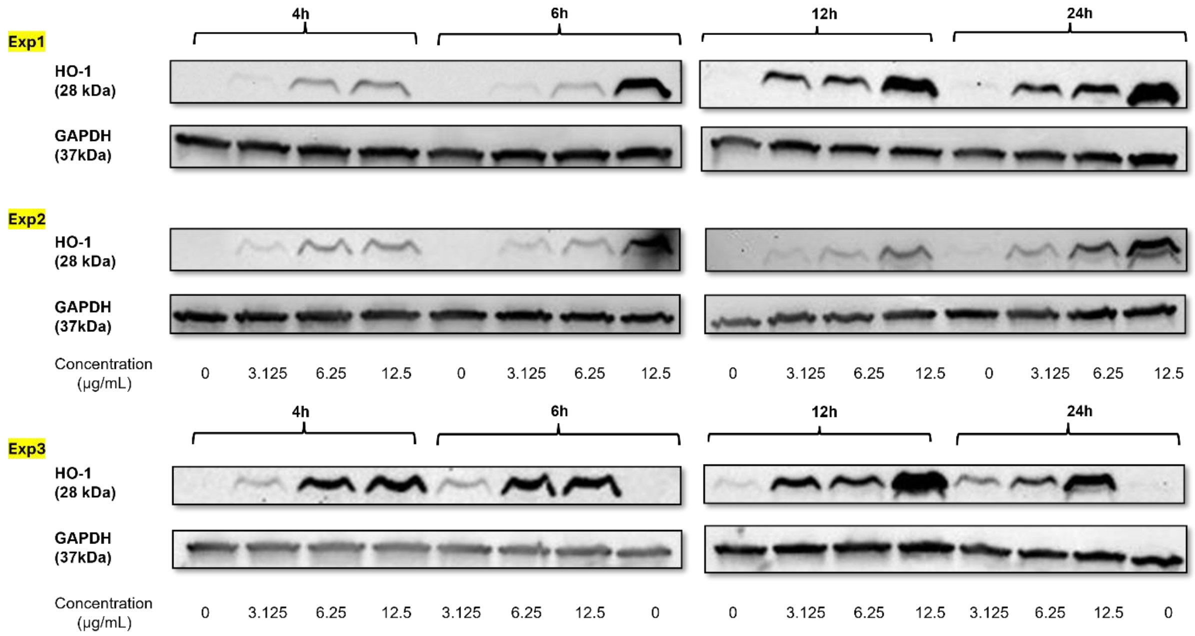

Figure S15: Cynaropicrin induces HO-1 protein expression in HepG2 cells

Relative protein expression of heme oxygenase-1 (HO-1) following the treatment of cynaropicrin (n=3 individual experiments). HepG2 cells were cultured in T75 flasks and exposed to negative control '0' (cells with medium only) and three concentrations of cynaropicrin (3.125, 6.25, & 12.5 µg/mL) for 4, 6, 12, or 24 h. GAPDH was used as the loading control.
